# Gene Expression Profiling and Fine Mapping Identifies a Gibberellin 2-Oxidase Gene Co-segregating With the Dominant Dwarfing Gene *Ddw1* in Rye (*Secale cereale* L.)

**DOI:** 10.3389/fpls.2019.00857

**Published:** 2019-07-03

**Authors:** Eva-Maria Braun, Natalia Tsvetkova, Björn Rotter, Dörthe Siekmann, Konrad Schwefel, Nicolas Krezdorn, Jörg Plieske, Peter Winter, Gilbert Melz, Anatoly V. Voylokov, Bernd Hackauf

**Affiliations:** ^1^Institute for Breeding Research on Agricultural Crops, Julius Kühn-Institut, Quedlinburg, Germany; ^2^Department of Genetics and Biotechnology, Saint Petersburg State University, Saint Petersburg, Russia; ^3^GenXPro GmbH, Frankfurt am Main, Germany; ^4^HYBRO Saatzucht GmbH & Co. KG, Schenkenberg, Germany; ^5^TraitGenetics GmbH, Gatersleben, Germany; ^6^Nordic Seed Germany GmbH, Nienstädt, Germany; ^7^Vavilov Institute of General Genetics Russian Academy of Sciences, Moscow, Russia

**Keywords:** plant height, lodging tolerance, comparative mapping, marker-assisted selection, genetic diversity, hybrid breeding

## Abstract

The gibberellin (GA)-sensitive dwarfing gene *Ddw1* provides an opportunity to genetically reduce plant height in rye. Genetic analysis in a population of recombinant inbred lines confirmed a monogenetic dominant inheritance of *Ddw1*. Significant phenotypic differences in PH between homo- and heterozygotic genotypes indicate an incomplete dominance of *Ddw1*. *De novo* transcriptome sequencing of *Ddw1* mutant as well as tall genotypes resulted in 113,547 contigs with an average length of 318 bp covering 36.18 Mbp rye DNA. A hierarchical cluster analysis based on individual groups of rye homologs of functionally characterized rice genes controlling morphological or physiological traits including plant height, flowering time, and source activity identified the gene expression profile of stems at the begin of heading to most comprehensively mirror effects of *Ddw1*. Genome-wide expression profiling identified 186 transcripts differentially expressed between semi-dwarf and tall genotypes in stems. In total, 29 novel markers have been established and mapped to a 27.2 cM segment in the distal part of the long arm of chromosome 5R. *Ddw1* could be mapped within a 0.4 cM interval co-segregating with a marker representing the C20-GA2-oxidase gene *ScGA2ox12*, that is up-regulated in stems of *Ddw1* genotypes. The increased expression of *ScGA2ox12* observed in semi-dwarf rye as well as structural alterations in transcript sequences associated with the *ScGA2ox12* gene implicate, that *Ddw1* is a dominant gain-of-function mutant. Integration of the target interval in the wheat reference genome sequence indicated perfect micro-colinearity between the *Ddw1* locus and a 831 kb segment on chromosome 5A, which resides inside of a 11.21 Mb interval carrying the GA-sensitive dwarfing gene *Rht12* in wheat. The potential of *Ddw1* as a breeder’s option to improve lodging tolerance in rye is discussed.

## Introduction

Winter rye (*Secale cereale* L.) contributes to crop diversity primarily in a belt that ranges from Northern Germany through Scandinavia, the Baltic states, Poland, Ukraine, and Belarus, into central and northern Russia. This multipurpose small grain cereal is highly productive particularly as hybrid variety due to its efficient and sustainable use of nutrients and water. Rye may contribute to counteract the global trend of species-poor food supply (Khoury et al., 2014) because of beneficial effects of rye on health (Jonsson et al., 2018). The cultivation of rye supports compulsions to act in modern agriculture under the directive of the multiple challenges concerning global food security (Lal, 2016).

Like in other cereals, yield and quality of rye may be negatively affected by lodging, which refers to the displacement of stems from an upright position. Plant height (PH) is a major factor influencing lodging tolerance and counts among the major target traits in rye breeding (Geiger and Miedaner, 2009). PH is a quantitative inherited trait with complex genetic architecture in rye (Miedaner et al., 2011, 2012, 2018; Auinger et al., 2016). Although hybrid breeding resulted in significant breeding progress in rye (Laidig et al., 2017), the genetic gain achieved for lodging tolerance of this cereal crop during the last 26 years has been marginal (Laidig et al., 2018).

The introgression of major dwarfing genes has proven to be one of the most successful breeding strategies to promote lodging resistance of cereals (Hedden, 2003). In rye, several mutants carrying Gibberellin (GA)-sensitive and GA-insensitive dwarfing genes are known (Börner et al., 1996). In GA-sensitive mutants, a tall phenotype can be restored by the exogenous application of GA. In contrast, GA-insensitive mutants reveal a reduced response or react complete insensitive to the application of GA.

The dominant dwarfing gene *Ddw1* is a spontaneous mutant (Kobyljanski, 1972) and provides an opportunity to genetically reduce PH in rye. *Ddw1* originates from the germplasm collection preserved at the Vavilov Institute of Plant Industry in St. Petersburg (Kobyljanski, 1972) and belongs to the group of GA-sensitive dwarfing genes (Börner and Melz, 1988). *Ddw1* is described to improve PH in open pollinating population varieties in Eastern European (Kobyljanski, 1988; Torop et al., 2003), Canadian (McLeod et al., 2000) and Scandinavian (Tenhola-Roininen and Tanhuanpää, 2010) rye breeding programs. Despite of the genetic gain that has been achieved concerning rye productivity in population varieties carrying *Ddw1* (McLeod et al., 2000; Torop et al., 2003), the potential of *Ddw1* has yet not been exploited in hybrid breeding programs (Miedaner et al., 2011, 2012, 2018). The precise identification of *Ddw1* by marker-assisted selection already at the seedling stage as well as a differentiation between homo- and heterozygous semi-dwarf genotypes would improve the efficiency of rye breeding and asks for molecular markers, which enable the precise introgression of *Ddw1* in elite inbred lines of the seed parent pool.

*Ddw1* has been mapped to a 17.1 cM interval between the RFLP marker *WG199* and the isozyme locus *ß-amy-R1* on the distal end of the long arm of chromosome 5R (Korzun et al., 1996). Quantitative analysis of PH identified a major QTL assigned to *Ddw1* within a 43.2 cM interval (Tenhola-Roininen and Tanhuanpää, 2010). A QTL with less pronounced effect on PH residing in a 5.9 cM segment on chromosome 5R was attributed to *Ddw1* as well (Miedaner et al., 2012). In Triticale, a major QTL for PH has been credited to *Ddw1* in a 1 cM interval on chromosome 5R based on DArT markers (Alheit et al., 2014; Liu et al., 2014; Würschum et al., 2014a,b). However, the map position of this QTL on the short arm of chromosome 5R in Triticale rather than on the long arm of chromosome 5R as described for rye challenges the chromosomal localization of *Ddw1*.

Here, we report on the development of novel PCR-based markers closely linked to *Ddw1*. For this purpose, we have (i) analyzed PH in two bi-parental mapping populations segregating for *Ddw1*, (ii) performed gene expression profiling between bulks of defined *Ddw1* genotypes by Massive Analysis of cDNA Ends (MACE) for marker development, (iii) mapped novel markers in the two independent mapping populations segregating for *Ddw1*, and (iv) integrated the *Ddw1* target interval in a high density map of rye for comparative mapping. Furthermore, we used the recently released rye draft genome sequence (Bauer et al., 2017) to depict genes involved in gibberellin biosynthesis and signaling as putative candidate genes for *Ddw1*.

## Materials and Methods

### Plant Materials

The population A of recombinant inbred lines (RIL) originated from the cross R1620 (wild-type, tall) and R347/1 (mutant, semi-dwarf). This material was advanced to nine *F*_4__:__5_ families (*N* = 697) derived from heterozygous *Ddw1* genotypes of a segregating *F*_3__:__4_ family (Supplementary Figure 1). In addition, two genotypes of the Peterhof rye stock collection, the inbred line l.7 (tall, smooth peduncle, green leaf base) and p. 83 (dwarf – carrying *Ddw1* as well as (i) the GA-insensitive dwarfing gene compactum 2 (*ct2*), (ii) hairy peduncle (*Hp*), and (iii) purple leaf base (*An5*, Melz and Thiele, 1990), were crossed. One single F1 plant from this cross, heterozygous for *Ddw1*, *ct2*, *Hp*, and *An5*, was used to produce 101 F2 plants (population B) for genetic analyses and mapping purposes.

The RIL population L2039-N x DH (Martis et al., 2013) as well as disomic wheat-rye addition lines kindly provided by Steve Reader (Department of Crop Genetics, John Innes Centre, Norwich, United Kingdom) were used for chromosomal localization of *Ddw1* markers.

### Phenotypic Data Analyses

Plant height of population A was assessed on single plants at the Julius Kühn-Institut experimental station in Gross Lüsewitz, Mecklenburg-Western Pomerania, Germany, while the phenotyping of population B was performed at the experimental field of the St. Petersburg State University, Russia. The height of the individual plants was measured at BBCH87 from the soil surface to the end of the main stem. The red leaves base in the F2 population was scored at the time of plant heading, while the hairy peduncle was assessed after heading of all tillers. Analysis of variance (ANOVA), *post hoc* Tukey’s honest significant difference (HSD) test and plotting were performed with standard functions of the R statistical environment (R Core Team, 2017) and the R package agricolae (de Mendiburu, 2017). The average dominance effect ā of *Ddw1* on PH was estimated according to Comstock and Robinson (1952). The magnitude of ā measures the degree of dominance in the action of *Ddw1* as follows: ā = 0: no dominance, 0 < ā < 1: incomplete dominance, ā = 1: complete dominance, and ā > 0: overdominance.

### Identification of GA Metabolic and Signaling Genes in Rye

Rye genes involved in the GA biosynthesis and signaling pathway were filtered from the draft genome sequence of rye based on the predicted annotation (Bauer et al., 2017) and complemented by BLAST (Altschul et al., 1990) comparisons with a similarity cut-off of >80% for rice (*Oryza sativa* L.) sequences (Hirano et al., 2008; Huang et al., 2015) and >90% for wheat (*Triticum aestivum* L.) sequences (Pearce et al., 2015; Clavijo et al., 2017) as queries and an alignment length ≥ 100 bp. The available information on protein-coding genes in rye was complemented using the AUGUSTUS software (Stanke et al., 2008) to predict genes on Lo7 contigs. Orthologous GA genes from barley, *Brachypodium distachyon*, rice and wheat were downloaded from the Ensembl Plant Database release 41 (Aken et al., 2017). Peptide sequences of GA2-oxidase genes AT1G78440.1 (*AtGA2ox1*), AT1G30040.1 (*AtGA2ox2*), AT2G34555.1 (*AtGA2ox3*), AT1G47990.1 (*AtGA2ox4*), AT1G02400.1 (*AtGA2ox6*), AT1G50960.1 (*AtGA2ox7*), and AT4G21200.1 (*AtGA2ox8*) from *Arabidopsis thaliana* were downloaded from the Phytozome database (Goodstein et al., 2012) and aligned together with peptide sequences from *Brachypodium dystachion*, rice, wheat, barley, and rye using the MUSCLE algorithm implemented in MEGA6 (Tamura et al., 2013). The phylogenetic tree was reconstructed with MEGA6 using the Maximum Likelihood method and the reliability of the tree topology was validated using the bootstrap method with 1000 replicates.

### Gene Expression Profiling in Rye

*De novo* transcriptome sequencing was performed using a modified RNA-seq variant, Massive Analysis of cDNA Ends (MACE). MACE generates one sequence per transcript preferentially from the 3′-end of the respective cDNA. Thus, contigs assembled from these sequences usually represent the 3′-UTR of the gene. Coverage of this target region is sufficiently high to reliably identify SNPs even in lowly abundant transcripts. For MACE, each of 96 *F*_6:7_ RIL (Supplementary Figure 1) were included in either normal or semi-dwarf near-isogenic bulks (NIB). For a comprehensive characterization of the rye transcriptome, total RNA was isolated from both NIB established from six different tissues collected at five developmental stages (i.e., BBCH stage, Lancashire et al., 1991). The following tissues were harvested at distinct developmental stages: roots and caryopses as well as coleoptiles after germination (BBCH 7), leaves when two leaves were unfolded (BBCH 12), stems at the end of tillering (BBCH 29), during stem elongation (BBCH 37) and at begin of heading (BBCH 51), and ears when inflorescence emerged (BBCH 51). RNA was isolated using the NucleoSpin^®^ miRNAkit (Macherey Nagel, Düren, Germany), which allows to separate the large and small fraction of the total RNA. The fraction of the total RNA > 200 bp and the small RNA were isolated and used separately for the preparation of the MACE libraries. The quality of the RNA samples was analyzed running the electrophoretic assay “Plant RNA nano” on an Agilent2100 Bioanalyzer (Agilent Technologies, Santa Clara, CA, United States). The preparation of MACE libraries, the sequencing using an Illumina Hiseq2000 (Illumina Inc., San Diego, CA, United States) with 1 × 100 bps and the quantification of mRNA expression was performed according to Zawada et al. (2014). The assembly of contigs from single MACE sequencing reads was done using the Trinity RNA-Seq *de novo* Assembly (Version: trinityrnaseq_r20140717, Grabherr et al., 2011).

The Relative Log Expression (RLE) method implemented in the R-package DESeq2 (Love et al., 2014) was used for data normalization. Principal component analysis (PCA) for all expression profiles in the six rye organs used, as well as the detection of differentially expressed genes (DEG) between the *Ddw1* mutant and the wild type were performed with DESeq2. Transcripts were considered as significantly differentially expressed between mutant and wild type at an adjusted *p*-value threshold (Benjamini and Hochberg, 1995) of < 0.05 and a log2 fold change (log2FC) threshold of *x* ≥ 2 (up-regulated) or *x* ≤ −2 (down-regulated). Visualization of the DEGs was done with DESeq2 after regularized logarithm transformation of the raw read counts and *z*-scores were used to compare significant changes in gene expression including fold changes.

For annotation of the MACE contigs these were BLASTed against two rye transcriptome assemblies (Haseneyer et al., 2011; Khalil et al., 2015), rye whole chromosome arm (WCA) shotgun sequences (Martis et al., 2013), the Lo7 whole genome shotgun (WGS) contigs (v2) as well as Lo7 gene models (v3) (Bauer et al., 2017), barley (*Hordeum vulgare* ssp. *vulgare*) full length cDNAs (Sato et al., 2009; Matsumoto et al., 2011), coding sequences of *Aegilops tauschii* (Jia et al., 2013), *Triticum urartu* (Ling et al., 2013), barley (Mascher et al., 2017) and wheat (IWGSC, 2018), as well as *Brachypodium distachyon* (Brachypodium genome annotation v1.2^1^ and rice (*Oryza sativa* subsp. *japonica*) proteins. Matching rice sequences were used to integrate the information from the Q-TARO database on cloned and functionally characterized rice genes (Yonemaru et al., 2010). The number of matching sequences were counted after filtering according to the best hit display with a similarity ≥80% (BLASTX ≥ 75%) and an alignment length ≥ 100 bp (BLASTX ≥ 30 amino acids). Annotation of yet un-assigned rye transcripts was performed based on sequence similarity to other sequenced species using NRPEP, the NCBI non-redundant protein sequences database, with an *e*-value threshold of the BLASTX search set to 1e–20.

### Development and Mapping of Novel *Ddw1* Markers

PCR primer were derived from rye transcripts as previously described (Hackauf et al., 2009, 2012) and supplemented by primers established from mapped wheat ESTs (Ishikawa et al., 2009; Akhunov et al., 2010). The SNPs in the MACE contigs comp31371 and c10806 were assayed based on the tetra-primer ARMS–PCR procedure (Ye et al., 2001). Detailed information on assay conditions and primer sequences of the markers are given in the Supplementary Table 1. Multipoint linkage analysis of markers was performed using the software package JoinMap v.4.0 (Van Ooijen, 2006). The Kosambi function was applied to convert recombination values to genetic distances (cM). The recombinant inbred line population L2039-NxDH (Martis et al., 2013) was genotyped using a 15k Infinium SNP array, which was derived from the 90k iSELECT SNP-chip described by Wang et al. (2014). Development and genotyping of the 15k SNP-chip was performed at TraitGenetics GmbH^2^. The high-density linkage map for chromosome 5R was established through the QTL IciMappingV3.2 software using the nnTwoOpt algorithm for ordering and the Sum of Adjacent Recombination Frequencies (SARF) with a window size of eight markers as criterion for rippling (Meng et al., 2015). Graphical presentation of the established maps was performed using MapChart (Voorrips, 2002).

## Results

### Phenotyping and Segregation Analysis

**FIGURE 1:**
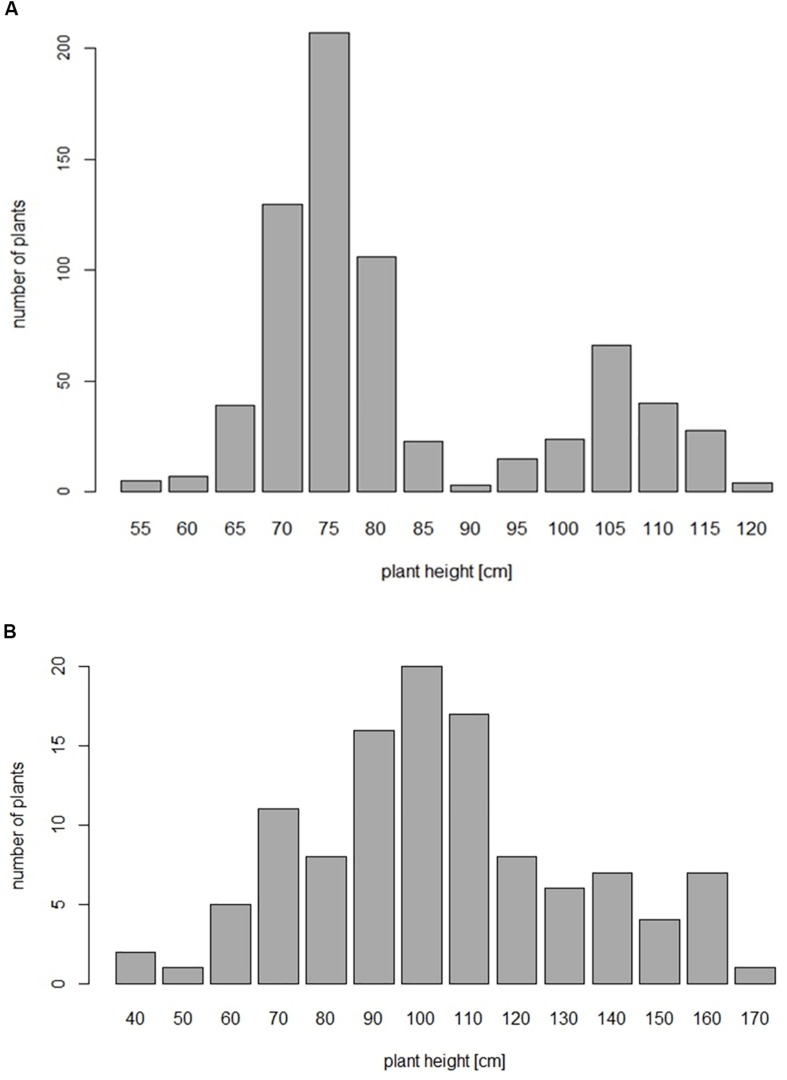
**(A)** Frequency distribution of phenotypic data for plant height in *F*_4__:__5_ progenies (*N* = 697) from the cross “R1620” (*ddw1*) × “R347/1” (*Ddw1*). **(B)** Frequency distribution of phenotypic data for plant height in a F2 population from the cross “l.7” (*ddw1*) × “p. 83” (*Ddw1*).

**FIGURE 2:**
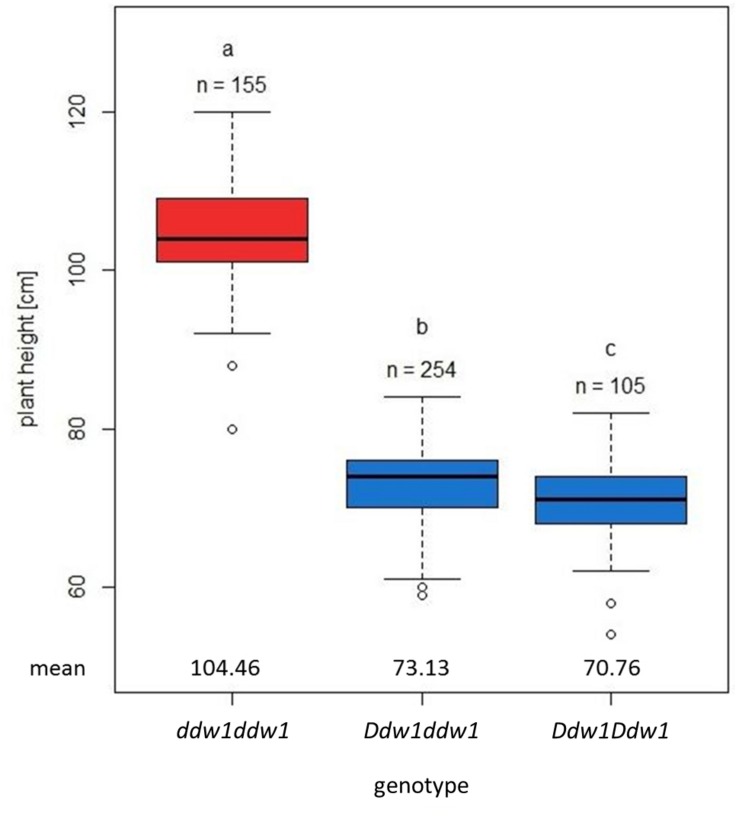
Effect of *Ddw1* on plant height in *F*_4__:__5_ near isogenic lines of the cross “R1620” (*ddw1*) × “R347/1” (*Ddw1*). Boxplots show the median (horizontal line), upper and lower quartile, and whiskers (vertical bars) of homo- (*Ddw1Ddw1*) and heterozygous (*Ddw1ddw1*) semi-dwarf as well as homozygous tall (*ddw1ddw1*) genotypes. The genetic constitution at the *Ddw1* locus was derived based on two co-dominant flanking markers, *tcos4366* and *c28517*. Different indices between *Ddw1* genotypes designate significantly (*p* < 0.05) different means (Tukey’s HSD Test).

The 697 plants segregating for PH in population A revealed a bimodal distribution (Figure 1A) and could be classified in two groups, either shorter or taller than 85 cm. The observed segregation in 517 semi-dwarf and 180 tall plants did not significantly deviate (*p* < 0.05) from a 3:1 ratio between mutant and wild type plants (Supplementary Table 2). *Ddw1* genotypes were deduced based on two co-dominant flanking markers, *tcos4366* and *c28517*, respectively (*cf*. linkage analysis). RIL homozygous for the wild-type *ddw1* allele were significantly taller than the height-reducing *Ddw1* carriers (*p* < 0.05) with the heterozygous entries not in between the two homozygous classes (Figure 2). As compared to the wild type, PH is reduced on average by 33.69 cm (32.3%) in homozygous semi-dwarf genotypes, while height reduction in heterozygous genotypes is slightly less pronounced (−30.0%, Figure 2). The statistical significant (*p* < 0.05) difference in PH between homozygous and heterozygous *Ddw1* carriers was on average 2.37 cm, the average degree of dominance for *Ddw1* on PH is ā = 0.86.

In population B, the phenotypic data for PH followed a normal distribution (Figure 1B). *Ddw1* and *ct2* have been assessed based on pleiotropic effects of each gene. Plants carrying *Ddw1*, in addition to a shorter stem, have delayed heading and wider leaves compared to wild type plants. Plants homozygous for *ct2* are characterized by erect leaves and nearly awnless ears. The latter phenotype, together with third flowers and short round kernels, result in an ear morphology similar to that of wheat. The segregation in this population for *Ddw1* (72:18, χ^2^_0.05,1_ = 1.2) as well as *ct2* (63:27, χ^2^_0.05,1_ = 1.2), *Hp* (67:29, χ^2^_0.05,1_ = 0.6), and *An5* (66:22, χ^2^_0.05,1_ = 0.0) fitted the expected 3:1 ratios for monogenic inheritance.

### Identification of Gibberellin Biosynthesis and Signaling Genes in Rye

The recently released draft of the rye genome sequence comprises a set of 78 genes encoding the GA biosynthesis and signaling pathway (Bauer et al., 2017; Supplementary Table 3). The shotgun survey sequences of flow-sorted rye chromosomes (Martis et al., 2013) covered 59 (75.6%) of these genes. The four enzymes of the early steps of GA biosynthesis, that is to say *ent*-copalyl diphosphate synthase (CPS), *ent*-kaurene synthase (*KS*), *ent*-kaurene oxidase (*KO*), and *ent*-kaurenoic acid oxidase (*KAO*), have been predicted to be encoded by six, eight, one and six genes, respectively. Likely orthologs of 2-oxoglutarate-dependent dioxygenases (2-ODD) governing the subsequent steps of GA biosynthesis and inactivation have been predicted in the genomic survey data from rye inbred line Lo7 as well. Based on the nomenclature suggested by Pearce et al. (2015), seven GA20-oxidase (*GA20ox*) genes, five *GA3ox* genes and 14 *GA2ox* genes could be indexed in rye. Furthermore, the catalog of rye GA biosynthesis genes contains *ScGA1ox1*, an ortholog of *TaGA1ox-B1* (Pearce et al., 2015) as well as both likely rye orthologs of the rice *GA13ox* genes. Likewise, five structural genes encoding cytochrome P450 monooxygenases, including the ortholog of the rice Elongated uppermost internode (*EUI*) gene, represent a pathway for GA deactivation in rye. Rye harbors a family of eight genes corresponding to the GA receptor *GID1* (GA-INSENSITIVE DWARF1). A key event in GA signaling is the degradation of DELLA proteins, which are negative regulators of the GA response that interact with GID1 in a GA-dependent manner (Schwechheimer, 2012). We could slightly extend the set of already predicted 75 gene models (Bauer et al., 2017) by three gene models including *ScSLR1*. *ScSLR1* represents a rye homolog of DELLA proteins, a family of transcriptional factors encoding height-regulating genes, such as *Rht-B1* and *Rht*-*D1* in wheat, *D8* in maize, *GAI* and *RGA* in *Arabidopsis* as well as *SLR1* in rice (Ikeda et al., 2001). *ScSLR1* maps to the short arm of chromosome 4R in rye while three paralogous genes encoding DELLA proteins in rye are located on chromosome 1R and 2R, respectively. The set of rye GA signaling genes includes the ortholog of the rice GA-insensitive dwarfing gene *D1*, four *GAMYB* transcription factors as well as the orthologs of the GA signaling regulators SPINDLY (*SPY*), SNEEZY (*SNY*), EARLY FLOWERING1 *(EL1)* as well as three protein kinases associated with GAMYB (*KGM*). For a subset of 26 (32.1%) GA biosynthesis and signaling genes we have validated the chromosomal localization via disomic wheat-rye addition lines, 12 genes could be integrated in the L2039-NxDH map. The primers derived from the rye ortholog of the rice floral repressor *early flowering1*, a key regulator of the GA response (Dai and Xue, 2010), amplified a paralog residing on chromosome 4RL in the L2039-NxDH map. This paralog is neither represented in the whole chromosome arm shotgun sequences nor in the rye draft genome sequence. Sixty eight (83.9%) of the rye GA genes, are represented on the rye 600k array with up to six single nucleotide variants (Supplementary Table 3). In total, four GA biosynthesis genes (*ScKAOL4*, *ScKAOL5*, *ScGA3ox6*, *ScGA2ox12*) and six GA signaling genes (*ScEUIL2*, *ScEUIL4*, *ScEUIL3*, *ScD1*, *ScGIDL2*, *ScEL1*) map to chromosome 5R and might, thus, serve as candidates for *Ddw1*.

### Expression Profiling

**FIGURE 3:**
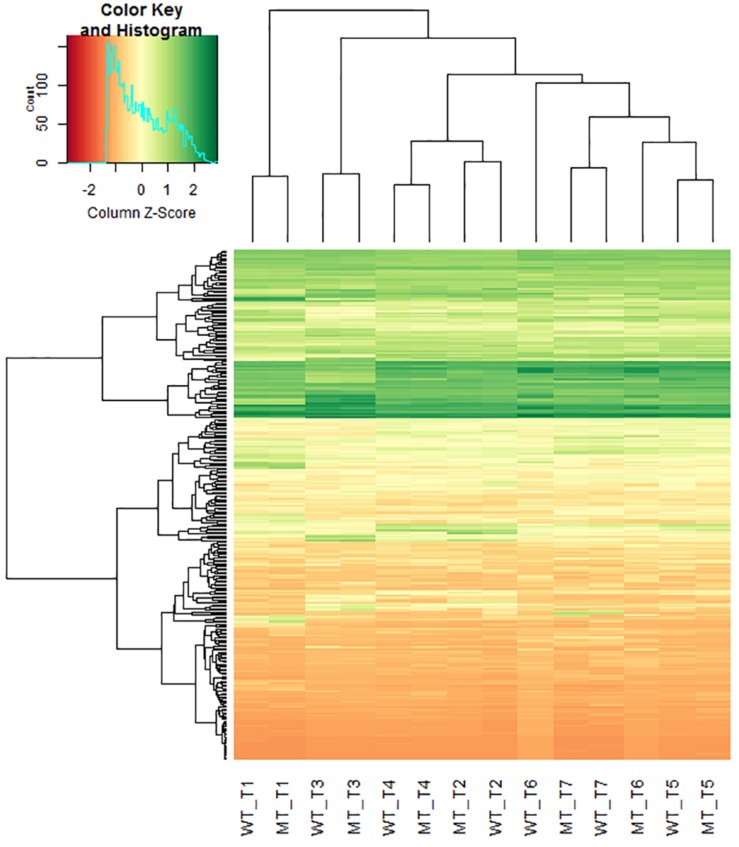
Hierarchical clustering heatmap of rye homologs of rice genes controlling plant height in different tissues of semi-dwarf and tall rye genotypes. Each row represents a contig and each column represents a defined tissue and genotype. The *z*-score of fold-change values for each sample were subjected to hierarchical clustering using standard correlation. The color scale ranges from negative (red) to positive *z*-scores (green) explaining the deviance of mean expression. WT, normal NIB; MT, semi-dwarf NIB; T1, Root and caryopses EC07; T2, Coleoptile EC07; T3, Leaf EC12; T4, Stem EC29; T5, Stem EC37; T6, Stem EC51; T7, Ear EC51.

*De novo* transcriptome sequencing of *Ddw1* mutant and tall NIBs enabled to assemble 113,547 contigs with an average length of 318 bp covering 36.18 Mbp (Supplementary Table 4). Of these, 75,087 (66.1%) contigs were anchored via BLASTN to the 2.8 Gbp rye genome sequence including 24,815 contigs (21.9%) mapping to a defined position in the rye high-density map. A total of 67,485 (59.4%) contigs matched shotgun survey sequences of flow-sorted rye chromosomes, and 40,554 (35.7%) could by unambiguously assigned to rye chromosomes 1R (5,811; 5.1%), 2R (6,711; 5.9%), 3R (6,069; 5.3%), 4R (6,940; 6.1%), 5R (7,587; 6.7%), 6R (5,789; 5.1%), as well as 7R (1,647; 1.5%). The dataset was further indexed using two collections of rye cDNA (38.1 and 50.8% hits, respectively), coding sequences of *T. aestivum* (A genome: 29,994, 26.4%; B genome: 29,777, 26.2%; D genome: 26,962, 23.7%), *Ae. tauschii* (22,847, 20.1%), and *T. urartu* (22,187, 19.5%), full-length cDNA of barley (27,543, 24.3%), as well as the barley reference genome (26,499, 23.3%). A total of 430 homologous and functionally characterized rice genes were hit by 759 MACE sequences (Supplementary Table 5). These genes control morphological or physiological traits including PH (95 rice genes/ 176 rye transcripts), flowering time (22/47) and source activity (54/115), or confer resistance to abiotic stress like drought (43/75) or low temperature (25/30). The position in the Lo7xLo225 high-density map was available for 225 (52.3%) of these genes. We have performed a hierarchical cluster analysis to investigate effects of *Ddw1* on the expression pattern of these genes. In general, the gene expression profiles in different tissues from semi-dwarf and tall rye are not influenced by *Ddw1* and tend to cluster together. However, the profiles of genes governing germination (Supplementary Figure 2) as well as the development of seedlings and shoots (Supplementary Figure 3) separate the expression profiles of coleoptiles from semi-dwarf and tall rye. Furthermore, *Ddw1* regulates the expression of genes controlling source/sink activity (Supplementary Figure 4) and flowering (Supplementary Figure 5), which is indicated by the separation of the gene expression profiles in coleoptiles and stems at begin of heading from both rye genotype bulks. A prominent difference between semi-dwarf and tall rye could be observed for the expression signature of genes affecting PH in stems at the beginning of heading (Figure 3). In total, the position in the Lo7xLo225 map is available for 48 rye orthologs to genes controlling PH in rice and eight of these PH genes (*Scbc1l4*, *Scgdd1*, *Scj10gBTF3*, *ScLTG1*, *ScPIL1*, *ScTid1*, *ScUgp1*, and *ScZHD1*) are residing on chromosome 5R (Supplementary Table 5).

The comparison between the rye high-density map and shotgun survey sequences of flow-sorted rye chromosomes mapped 8,150 (7.2%) of the EST assemblies described here unambiguously on chromosome 5R. Comparative mapping between the distal part the long arm of rye chromosome 5R and barley chromosome 4H indicated, that the colinearity of both subgenomic regions is interrupted by a short introgressed segment homeologous to barley chromosome 7H (Supplementary Figure 6). Two MACE contigs matching Lo7_v2_contig_2880798 and Lo7_v2_contig_233128, which are flanking the *Ddw1* genomic region on chromosome 5RL in the Lo7xLo225 high-density map, correspond to the barley gene models HORVU7Hr1G031190 and HORVU7Hr1G089960 on chromosome 7H, respectively (Supplementary Table 4). Further 4,498 contigs were excluded for marker development based on their position (i) on 5R in the Lo7xLo225 map as well as (ii) on barley chromosomes other than 4H and 7H, resulting in a final set of 3,652 (3.2%) 5R contigs. In 60 (0.1%) of these contigs single nucleotide polymorphisms between the bulks of mutant and wildtype plants were observed.

**FIGURE 4:**
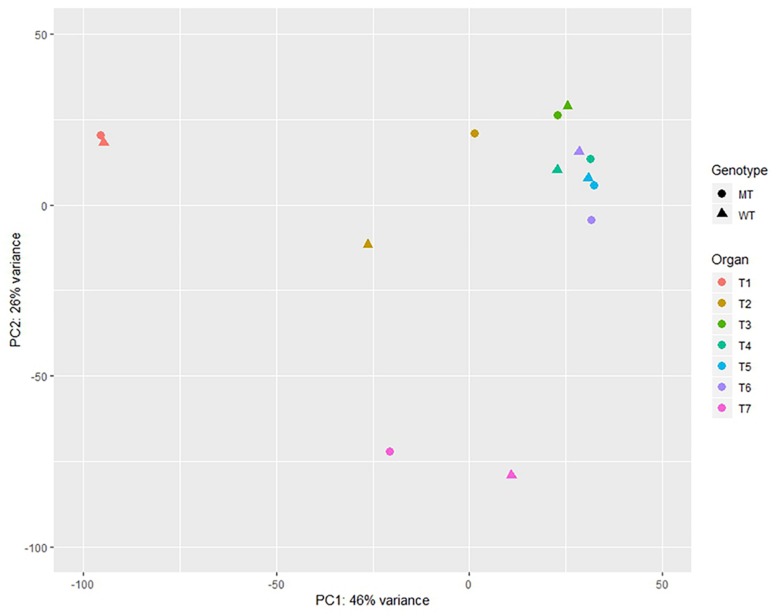
Principal component analysis of the expression data of normal and semi-dwarf near isogenic bulks (NIB) in different organs. WT, normal NIB; MT, semi-dwarf NIB; T1, Root and caryopses EC07; T2, Coleoptile EC07; T3, Leaf EC12; T4, Stem EC29; T5, Stem EC37; T6, Stem EC51; T7, Ear EC51.

A principal components analysis (PCA) illustrated, that the samples largely formed distinctive clusters based on their tissue sources when projected on the two first principal components (Figure 4). The most distinct groups are the roots and caryopses as well as ears when compared to the coleoptiles, leaves and stems. The stem samples segregate from the remaining samples, obviously based on key biological properties in this tissue. Within tissue variation due to genetic effects of *Ddw1* was low except for coleoptiles and ears as well as stems at heading.

**FIGURE 5:**
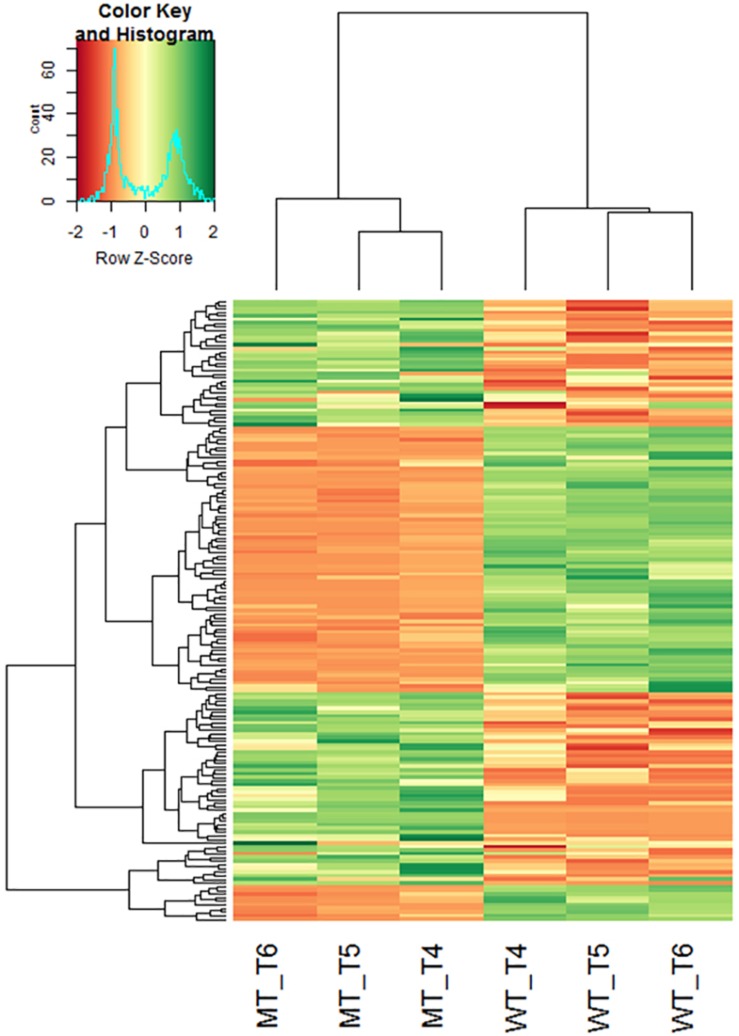
Hierarchical clustering heatmap of transcripts differentially regulated in stems of semi-dwarf and tall rye genotypes. Each row represents a contig and each column represents a defined tissue and genotype. The *z*-score of fold-change values for each sample were subjected to hierarchical clustering using standard correlation. The color scale ranges from negative (red) to positive *z*-scores (green) explaining the deviance of mean expression. WT, normal NIB; MT, semi-dwarf NIB; T4, Stem EC29; T5, Stem EC37; T6, Stem EC51.

**FIGURE 6:**
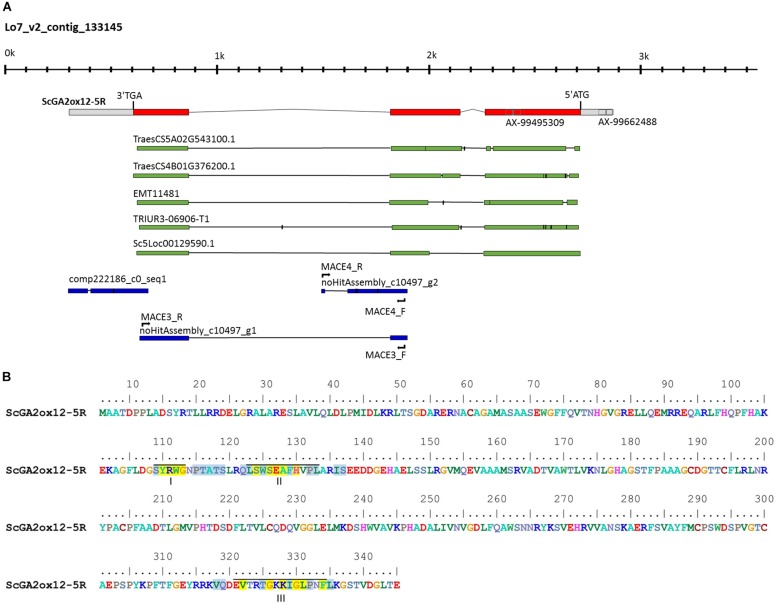
Characterization of the rye *GA2ox12* gene and the encoded gene product. **(A)** Structure of *ScGA2ox12* on the Lo7 contig 133145. The ATG start and TAG stop codons as well as the positions of two SNP markers of the rye 600k array are marked. Primer positions for the development of *Ddw1* markers are indicated. The alignments with cDNAs from *T. aestivum* (TraesCS5A02G543100 and TraesCS4B02G376200), *Aegilops tauschii* (EMT11481), *T. urartu* (TRIUR3-06906-T1), and *S. cereale* (Sc5Loc00129590.1) are given as green bars. Rye transcripts identified by expression profiling between semi-dwarf and tall near isogenic bulks are given in blue. **(B)** Predicted amino acid sequence of the *GA2ox12* gene product. Roman numerals below the sequences indicate the three unique and conserved motifs essential for function of C20 GA2oxs in controlling plant height according to Lo et al. (2017). Identical and conserved amino acid residues are highlighted in yellow and blue.

Differential gene expression analysis between the mutant and wild-type near isogenic bulks (NIB) was performed based on the three stem samples. Significant (*p* < 0.05) differences in gene expression between NIB were detected for 186 contigs (Supplementary Table 4). Inclusion of the fold change criterion reduced the number of differentially expressed genes (DEG) slightly to 171, with 88 genes up-regulated and 83 genes down-regulated in the semi-dwarf NIB (Figure 5). In total, 154 (87%) of the DEGs could be assigned to a rye chromosome and 19 (12.3%) of the DEGs were located on 5R. Homology based gene annotation detected a homeobox-leucine zipper protein, a DEAD-box ATP-dependent RNA helicase, a phospholipase, a glucosyltransferase (UGT) as well as a GA2-oxidase (GA2ox) gene among the DEGs on 5R. The UGT transcript comp52087_c0_seq1 is significantly down-regulated in semi-dwarf rye and could be anchored (97% identity, *E*-value 8.00 × E-153) to the 2.1 kb Lo7 rye genome survey contig 1361654. In contrast, the GA2ox transcript comp222186 is significantly up-regulated in semi-dwarf rye and could be anchored (92% identity, *E*-value 9.00 × E-159) to the 3.5 kb Lo7 rye genome survey contig 133145 (Figure 6A). A 2,565 bp gene was predicted on this contig encoding a 345 aa peptide sequence (Figure 6B). This rye protein contains the three conserved amino acid motifs ^S^/_P_YRWG, xS^W^/_V_SEA^F^/_Y_H^I^/_V_^P^/_I_^L^/_M_, and DVxxxGxKxGLxxF, which are keys for functions of C20 GA2-oxidases (Lo et al., 2017).

Two further MACE contigs, c10497_g1 (98%, 4.00E-112) and c10497_g2 (94%, 4.00E-124), map to Lo7 contig 133145 as well. The predicted gene structure was matched by the contig c10497_g1 only. The alignment of contig c10497_g2 revealed structural variation compared to contig c10497_g1 (Figure 6A) and suggested a further GA2ox copy not represented in the draft of the rye genome sequence. Noteworthy, contig c10497_g1 and the coding sequence predicted from Lo7 contig 133145 detected two orthologs in *T. aestivum*, TraesCS5A02G543100 and TraesCS4B02G376200 as well as two orthologs in *A. tauschii*, EMT11481 and EMT14198, respectively. EMT11481 and EMT14198 are represented by the gene model AET4Gv20853600 in the *A. tauschii* genome sequence (Luo et al., 2017). Contig c10497_g1 matched the wheat gene model TraesCS4D02G271300 as well. However, TraesCS4D02G271300 represents a paralog of this rye transcript, as it is located within a 7.55 Mb interval, which is defined by the gene models TraesCS4D02G267300 and TraesCS4D02G275100, respectively, and which corresponds to a segment at position 60.98 cM on chromosome 7R in the high-density map of rye (Supplementary Table 4).

**FIGURE 7:**
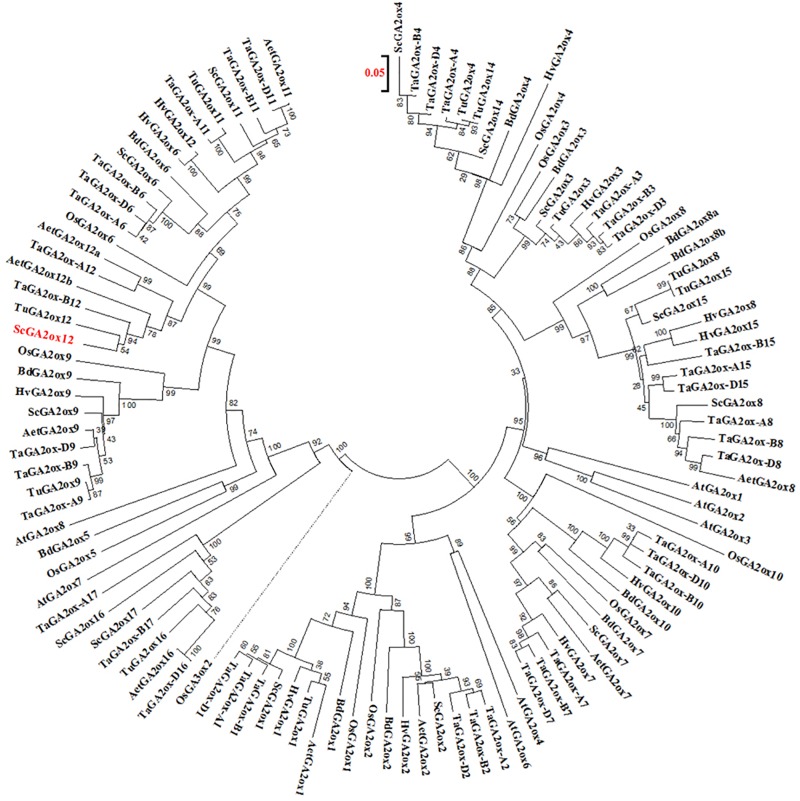
Phylogenetic relationships between GA2ox amino acid sequences from rye (Sc), bread wheat (Ta), barley (Hv), *Aegilops tauschii* (Aet), *Triticum urartu* (Tu), Brachypodium (Bd), rice (Os), and *Arabidopsis thaliana* (At). The sequence used as outgroup for the rooted tree is indicated by the dotted line. Bootstrap values are shown as percentage of 1000 replicates. The scale bare indicates the number of amino acid substitutions per site. The *GA2ox12* gene residing at the *Ddw1* locus in rye is given in red.

A phylogenetic analysis indicated that the GA2ox gene on Lo7 contig 133145 is closely related to the bread wheat gene *TaGA2ox-B12* (Figure 7). We have designated the differentially expressed rye GA2ox gene, thus, *ScGA2ox12*. Analogous we have named both *A. tauschii* orthologs *AetGA2ox12a* and *AetGA2ox12b*. The GA2ox genes fall into three paralogous clades each including the rice and *Brachypodium* paralogs *GA2ox3*, -*4*, -*7*, -*8*, and -*10* (clade I), *GA2ox1* and -*2* (clade II) and *GA2ox5*, -*6*, and -*9* (clade III). *ScGA2ox12* belongs to clade III and clustered, among others, together with *OsGA2ox6*, *TaGA2ox-A9*, and *TaGA2ox-A12*. The paralogous rye genes *ScGA2ox6*, *ScGA2ox9*, and *ScGA2ox11* of this clade map to rye chromosomes 2R, 6R and 7R, respectively. We identified four further GA2ox paralogs in the rye draft genome sequence, *ScGA2ox14*, *-15*, *-16*, and -*17. ScGA2ox14* maps to chromosome 1R and is most similar to *ScGA2ox4*. *ScGA2ox15*, *-16*, and -*17* clustered to clade III and are related to *OsGA2ox5* and *BdGA2ox5*. The chromosomal localization of the three rye genes has yet not been determined but can be predicted by synteny with wheat on chromosomes 1R and 3R.

### Linkage Analysis and Comparative Mapping of *Ddw1* in Rye, Wheat, and Triticale

**FIGURE 8:**
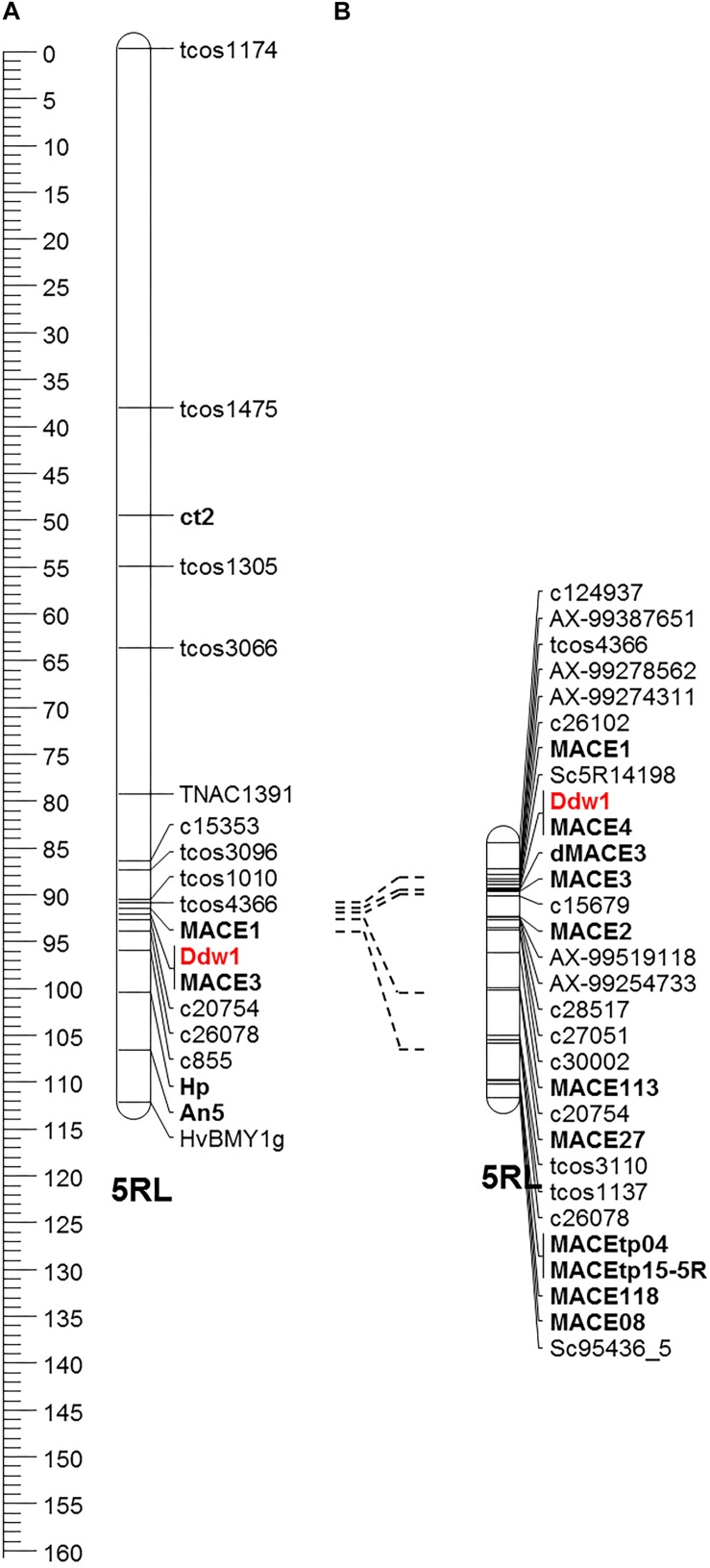
Fine mapping of *Ddw1* in rye. **(A)** Partial linkage map of chromosome 5R based on the cross “l.7 × p. 83” including the morphological genes *ct2*, *Ddw1*, *Hp*, and *An5*. **(B)** Partial linkage map of chromosome 5R including *Ddw1* based on 685 *F*_4__:__5_ progenies originating from the cross “R1620” × “R347/1.”

**FIGURE 9:**
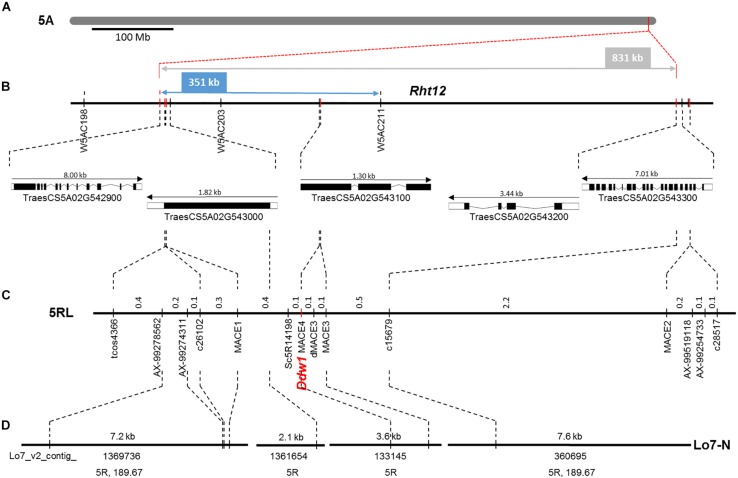
Comparative mapping of *Ddw1* in rye and *Rht12* in wheat **(A)** The position of the *Ddw1* orthologous segment on chromosome 5A of wheat is defined by the flanking markers c26102 and c15697 and indicated in red. **(B)** Integration of *Ddw1* markers, highlighted in red, in the *Rht12* interval. Orientation, length and designation of five predicted gene models residing in the 831 kb target interval is given below. **(C)** Partial linkage map of chromosome 5R including *Ddw1* based on 685 *F*_4:5_ progenies originating from the cross “R1620” × “R347/1.” The positions of markers are given in cM. **(D)** Integration of the *Ddw1* locus in the draft genome sequence of rye. The length and designation position of individual Lo7 rye genome survey contig is indicated. The positions of contigs represented in the high-density map of rye are given in cM. Common markers between individual maps are connected by dotted lines.

The macro-colinearity between the distal part of the long arm of chromosome 5R and *Brachypodium distachyon* chromosome Bd1 as well as rice chromosome R3 was used for the development of novel markers linked to *Ddw1*. This approach was complemented by rye transcripts identified in the present study as well as SNP markers from the high-density map of rye (Bauer et al., 2017). In the RIL population, 29 markers mapped to a 27.2 cM segment (Figure 8), that could be assigned to the long arm of chromosome 5R using disomic wheat-rye addition lines and amplicons obtained with the flanking *Ddw1* markers *tcos4366* and *tcos1137* (Supplementary Figure 7). The markers *AX-99387651*, *AX-99278562*, *AX-99274311*, *AX-99519118*, and *AX-99254733* allowed to integrate a 5.4 cM interval covering *Ddw1* in the high-density Lo7xLo225 map and supported the precise localization of *Ddw1* in this map at position 189.67 cM, as predicted by the flanking markers *c26102* and *c15679*, respectively (Figure 9). The marker *MACE3* was derived from contig c10497_g1 and discovered one recombination event among 685 defined *Ddw1* genotypes resulting in an estimated map position 0.2 cM distally of *Ddw1*. The marker *MACE4* was derived from contig c10497_g2 and co-segregated with *Ddw1* among 685 assayed genotypes. The map position of the dominant marker SC5R14198, which was developed based on EMT14198, was estimated 0.2 cM proximal from *Ddw1*. In population B, order and linkage relationships of the *Ddw1* markers have been successfully validated. In this population, the marker *MACE3* co-segregated with *Ddw1* among 90 *Ddw1* genotypes. The GA-insensitive dwarfing gene *ct2* mapped 38 cM proximal from *Ddw1*, while *Hp* and *An5* mapped 7.7 cM and 11.7 cM distal from *Ddw1*. Comparative mapping identified two segments on wheat chromosomes 5A and 4B, which are orthologous to the *Ddw1* locus in rye. The markers *c26102* and *c15679* are flanking *Ddw1* and define a 831-kb segment on wheat chromosome 5A, which resides within a 11.21 Mb interval carrying the GA-sensitive dwarfing gene *Rht12* in wheat (Sun et al., 2019) and which revealed perfect micro-colinearity to the *Ddw1* locus (Figure 9). In total, five genes have been predicted in the 831-kb segment including TraesCS5A02G543000, a UDP Glycosyltransferase (UGT) gene, and the *GA2ox* gene TraesCS5A02G543100. As described before, the *UGT* as well as the *GA2ox* gene are differentially expressed in stems of semi-dwarf and tall rye. In wheat, the marker *W5AC211* delimits a 483-kb segment in the *Rht12* interval proximal of a 10.73-Mb fragment deletion (Sun et al., 2019). The gene models TraesCS5A02G543000 and TraesCS5A02G543100 are located in the 531-kb region delimited by the rye marker *c26102* and *W5AC211*, respectively (Figure 9). The *Ddw1* orthologous segment on wheat chromosome 4B encompasses 3.24 Mb and carries 47 genes (Supplementary Table 7). This segment is characterized by an inversion relative to rye, which encompasses at least 1.27 Mb proximal of *Ddw1* as indicated by 15 genes within this 4B segment, which are represented in the recently published high-density of rye (Bauer et al., 2017; Supplementary Table 7). In order to validate the position of *Ddw1* in the rye genome, the novel markers were integrated in the genetic map established for the RIL population L2039-N x DH (Supplementary Figure 8). In this map, chromosome 5R covers 215 cM and is described by 159 DArT, 169 rye SNP, 68 STS as well as 30 wheat SNP markers (Supplementary Table 7). The orientation of this map was guided by the localization of 29 markers on the short and long arm of the homeologous group 5 chromosomes in wheat as well as by 15 STS markers assigned to the long arm of chromosome 5R using disomic wheat-rye addition lines. The *Ddw1* marker *MACE3* mapped 19.7 cM proximal of the microsatellite marker *Xscm312* at the distal end of the long arm of chromosome 5R and within a 37.4 cM segment defined by *Xgwm6* and the STS marker *HvBMY1g*, which was derived from a beta-amylase (*Bmy1*) gene of barley. A total of 102 previously mapped EST-based SNP markers (Martis et al., 2013) highlighted perfect colinearity between the L2039-N x DH and the integrated transcript map of rye. A comparison based on 46 DArT markers revealed an inverted orientation of chromosomes 5R in a Triticale map (Alheit et al., 2011) as well as in the Lo115-NxLo117-N map (Miedaner et al., 2012). The markers *rPt-507664* and *rPt-508266* defined a 6.7 cM target interval including *Ddw1* in the L2039-N x DH map as well as a 7.9 cM segment on chromosome 5R in Triticale including both QTL detected for PH and biomass yield in Triticale.

## Discussion

### A Breeder’s Option to Improve Plant Height in Hybrid Rye

A widespread use of *Ddw1* in rye breeding is currently hampered, as homo- and heterozygous semi-dwarf plants can phenotypically not be distinguished. The recessive allele for the tall growth habit is, thus, difficult to purify in open pollinating rye varieties, which may result in increasing frequencies of tall plants (McLeod et al., 2000; Tenhola-Roininen and Tanhuanpää, 2010). In rye, hybrid breeding is well established and offers a unique opportunity for a systematic integration of value-added traits. Heritable traits governed by single dominant genetic factors are expressed in homo- and heterozygous allelic states and, thus, particularly enable gene stacking in hybrids. Our genetic analyses confirm previous reports (Korzun et al., 1996; Tenhola-Roininen and Tanhuanpää, 2010) on a monogenic dominant inheritance of *Ddw1*. However, both previous genetic studies were conducted in F2 populations whereas we here analyzed a population of RIL, that originate from an independent F1 plant of the same cross as the F2 population analyzed by Korzun et al. (1996). Our strategy to systematically maintain heterozygosity for the *Ddw1* region in each generation of inbreeding is known as the heterogeneous inbred family (HIF) approach (Tuinstra et al., 1997) and has been successfully applied, e.g., for high resolution genetic and physical mapping of a major powdery mildew resistance QTL in barley (Hoseinzadeh et al., 2019). The residual heterozygosity comprising ca. 6% of the total genome in the F5 generation of our rye RILs reduces background noise as the process of consecutive selfing results in an average homozygosity of ca. 94%. Consequently, the bimodal expression of PH in our RIL clearly classified all single plants into semi-dwarf and tall phenotypes. In contrast, several F2 plants revealed an intermediate PH, probably caused by minor QTL (Korzun et al., 1996; Tenhola-Roininen and Tanhuanpää, 2010), which hampered an unequivocal phenotypic classification.

We describe the effects of *Ddw1* on line *per se* performance in PH and demonstrate, that heterozygote *Ddw1* genotypes display a phenotype which is not intermediate between the two homozygote phenotypes. Rather, heterozygous plants reveal a range of phenotypes close to homozygous short plants, with a statistical significant difference in PH between both genotypic classes indicating an incomplete dominance of *Ddw1* for PH. In rye breeding, target genotypes are highly heterozygous. In the RIL population, heterozygous *Ddw1* genotypes revealed a height reduction of 30%, which is stronger than height reduction associated with the GA-insensitive dwarfing gene *Rht-B1b* (−23%) in wheat but comparable to phenotypic effects of *Rht13* (−34%, Rebetzke et al., 2012), a GA-sensitive dwarfing gene located on wheat chromosome 7BS (Wang et al., 2015). For the GA-sensitive dwarfing gene *Rht12* residing on wheat chromosome 5AL a stronger phenotypic effect has been observed resulting in a height reduction of semi-dwarf *Rht12* lines ranging between 43−49% as compared to tall *rht12* genotypes (Sun et al., 2019). Testcross means for PH in highly heterozygous interpool rye hybrids ranged between 122 cm (Auinger et al., 2016) and 136 cm (Miedaner et al., 2018). Considering, that these data were influenced by the application of growth regulators, which is standard practice in western European cereal production and currently an indispensable factor in rye cultivation, *Ddw1* is expected to result in a moderate genetic reduction of PH in semi-dwarf rye hybrids. In wheat, single gene overdominance for grain yield has been reported, when the tall and dwarf parental genotypes were used to produce the F1 are respectively taller and shorter than an optimum in PH (Flintham et al., 1997). This result further encourages the integration of *Ddw1* in hybrid rye breeding programs aiming at the identification of genotype combinations with a genetically optimized PH. Furthermore, hybrid breeding permits a cutting edge technology to get rid of the recessive allele through the integration of *Ddw1* in seed parent lines. The production of semi-dwarf hybrids by crossing a stand of homozygous shorter male sterile plants with a stand of taller pollinator plants takes advantage of a height gap between parental genotypes to optimize the transfer of pollen. Thus, next to the recently established genome-based prediction of breeding values (Auinger et al., 2016), the integration of *Ddw1* in hybrid rye breeding programs offers a further option to genetically improve PH in rye.

### Deciphering GA Biosynthesis and Signaling Pathway Genes in Rye

Gibberellin biosynthesis and signal transduction genes have been proven to be valuable targets for the genetic improvement of PH in wheat and rice (Hedden, 2003). In the present study, the rye draft genome sequence (Bauer et al., 2017) served as a valuable resource to index a comprehensive set of genes governing the GA biosynthesis and signaling pathway in rye. These sequences facilitate further downstream applications including efficient development of gene specific primers as a prerequisite to systematically exploit rye germplasm collections for the identification of haplotypes not yet represented in elite inbred lines. The integrated genome scan and candidate gene analysis enabled a focused view on chromosome 5R and identified a set of GA genes for a targeted evaluation of their relationship to *Ddw1*, which belongs to the group of GA-sensitive dwarfing genes (Börner and Melz, 1988). The *ScGA2ox12* gene cosegregating with *Ddw1* is closely related to the 2β-hydroxylase *AtGA2ox8* from *A. thaliana*. Increased expression of *AtGA2ox8* has been shown to be responsible for dominant inherited dwarf phenotypes of activation-tagged *A. thaliana* lines (Schomburg et al., 2003). Heterologous expression of *AtGA2ox8* caused dwarfing in *Nicotiana tabaccum* (Schomburg et al., 2003) and *Brassica napus* (Zhou et al., 2011) as well. Transgenic *A. thaliana* lines overexpressing the transcription factor *GhMADS14*, which is specifically expressed in fibers of cotton (*Gossypium hirsutum*), revealed altered expression levels of genes related to the GA metabolic pathway including a significant up-regulation of *AtGA2ox8*, which resulted in a reduction in endogenous GA amounts in cells and GA-deficient phenotypes (Zhou et al., 2014). These observations indicate, that the increased expression of *ScGA2ox12* most probably may cause the dwarf phenotype of *Ddw1* genotypes, which would be consistent with the assumption, that *Ddw1* influences the GA biosynthesis pathway in rye. Noteworthy in this context, it is getting increasingly evident that the GA class of plant hormones is of pivotal relevance in the response of plants to abiotic stress (Colebrook et al., 2014). In rice, mutations in specific amino acids of *OsGA2ox6* moderately lowered GA levels and reprogrammed transcriptional networks, leading to reduced PH, more productive tillers, an expanded root system, a higher water use efficiency and photosynthesis rate, and elevated abiotic and biotic stress tolerance compared to wild type plants (Lo et al., 2017). The results of the GA2ox consensus tree analyses in the present study illustrate for the first time, that *OsGA2ox6* is closely related to *ScGA2ox12*. As phylogenetic relationships can provide information on gene function (Eisen, 1998), the results described for *OsGA2ox6* in rice stimulate further research to expand our understanding how *ScGA2ox12* operates in rye. This is particularly interesting with respect to practical rye breeding programs, as results on positive effects of the GA inhibitor paclobutrazol on PH, grain yield and drought tolerance in tef [*Eragrostis tef* (Zucc.) Trotter] and finger millet (*Eleusine coracana* Gaertn) recently revealed, that a genetically altered GA pathway is an attractive target for the breeding of climate-smart crop plants (Plaza-Wüthrich et al., 2016). In rye production, drought is a serious challenge as rye is mainly cultivated on light soils with low fertility and low water holding capacity. An average drought induced grain yield reduction of 23.8% has been reported for hybrid rye in non-irrigated compared to irrigated regimes under natural drought stress conditions (Hübner et al., 2013), while up to 57% grain yield reduction was observed in controlled environments under different drought regimes (Kottmann et al., 2015). The results described for *OsGA2ox6* in rice are congruent with the beneficial effects of the GA inhibitor paclobutrazol, which were described for tef and finger millet, and depict a genetic strategy to tackle the climate change by plant breeding. In the background of this knowledge *Ddw1* alters the GA content in a favorable manner and offers the opportunity to exploit natural diversity of genetic resources for hybrid breeding as novel approach to improve the agronomic performance of rye. The superior performance of population varieties carrying *Ddw1* in the drought prone Central Chernozem region of Russia (Torop et al., 2003) encourage to make use of *Ddw1* in central European rye breeding programs as well. These compiled aspects supplement previous judgements on the potential of major dwarfing genes like *Ddw1* for rye breeding (Miedaner et al., 2011, 2012). Despite of the promising prospects it needs to be considered that a comprehensive integration of *Ddw1* in hybrid breeding programs might narrow the genetic base of rye varieties. In order to counterbalance any potential linkage drag effect, which might be driven by strong selection for a single haplotype variant controlling PH, further GA-sensitive dwarfing genes like *Ddw2* (Melz, 1989), *Ddw3* (Stojałowski et al., 2015), or *Ddw4* (Kantarek et al., 2018) should be evaluated in breeding programs as well. The sequence information on rye GA2ox genes might support the integration of these valuable alleles in elite rye germplasm using precision breeding approaches. Hence, there is considerable potential in developing drought tolerant hybrid rye varieties based on an optimized GA homeostasis.

### Differential Gene Expression Reveals Candidate Genes for *Ddw1*

We report on the first comprehensive characterization of the gene expression pattern in rye plants carrying *Ddw1.* Considering the differential expression of the semi-dwarfing gene *sd-1* in normal-type and semi-dwarf rice (Monna et al., 2002) we hypothesized that genes responsible for the variation of gene expression between normal and semi-dwarf rye are responsible for the variation of PH as well. We thus assumed that the candidate gene governing *Ddw1* as the major genetic component of PH variation in the RIL population can be mined from the pattern of gene expression profiles.

The reduction of stem length is the most prominent phenotypic effect of *Ddw1*. We anticipated, that this biological feature is directly reflected by the transcript pattern of semi-dwarf compared to tall rye genotypes. Gene expression profiling in the stem was, thus, the main objective in our study. We complemented stem tissues with roots, caryopses, coleoptiles, leaves, and ears to capture a comprehensive part of the rye transcriptome. Hierarchical cluster analysis suggests spatial and temporal effects of *Ddw1* on gene expression in coleoptiles as well as in stems at the beginning of heading. Several independent sets of genes were responsible for the observed substructures. In coleoptiles the effects of *Ddw1* on the expression of genes involved in germination as well as the separation of this tissue from other clusters in the PCA reflects the central role of GA in mediating the developmental control of seed germination, and how this differs from vegetative growth processes (Urbanova and Leubner-Metzger, 2016).

The harvest index describes the ratio of harvested grain to total shoot dry matter (Donald and Hamblin, 1976) and represents the result of plant efficiency including a range of processes governing the packaging, transport and deposition of photoassimilates and nutrients into the seed (Smith et al., 2018). The introgression of dwarfing genes resulted in an improved harvest index as the most important change in the architecture of rice and wheat varieties that was responsible for increasing their yield potential in the course of the “Green Revolution” (Khush, 1995). Thus, the observed differences between semi-dwarf and tall rye in the expression pattern of genes controlling source/sink activity in spikes at heading fits to the expectation, that the balance between assimilation capacity (source) and grains (sink) is different in semi-dwarf and tall rye with respect to assimilate partitioning. *Ddw1* has been reported to affect the number of grains per spike, thousand grain weight, ear yield and flowering time (Börner et al., 1999), the latter of which is mirrored by the expression pattern of flowering genes. The observed differences in gene expression between semi-dwarf and tall rye especially in the stem samples at heading by an array of genes controlling PH corresponds with the phenotypic effects of *Ddw1* on PH.

The distribution of DEG in the rye genome is attributed to the genetic structure of the analyzed plant materials and reflects genetic diversity between NIB beyond the *Ddw1* locus on chromosome 5R, as could be demonstrated for a segment on chromosome 4R (data not shown). However, by combining bioinformatics and genetic mapping, DEG analysis successfully identified transcripts linked to *Ddw1*. To summarize, the clustering described in the present study confirmed our hypothesis on the stem as prime target for *Ddw1* effects on PH and, thus, for our gene expression profiling approach and may direct further research on *Ddw1*.

### Fine Mapping of *Ddw1*

Next generation sequencing (NGS) technologies have considerably accelerated the discovery of the genetic basis responsible for a phenotype in model (James et al., 2013) and crop plants (Mascher et al., 2014; Pankin et al., 2014; Hoseinzadeh et al., 2019). In these studies, high-quality genome sequences served as reference to guide the alignment of reads from whole-genome sequencing of bulked recombinants and a subsequent analysis for local skews in the parental allele frequencies (James et al., 2013). Results described in the present study illustrate constraints which currently limit the application of mapping-by-sequencing in rye. The *GA2ox12* gene co-segregating with *Ddw1* is neither represented in the high-density map nor in the improved linear gene order model of the recently described draft genome sequence of rye (Bauer et al., 2017). Likewise, synteny-based mapping-by-sequencing based on the high-quality barley genome sequence (Mascher et al., 2017) suffers from rearrangements between rye and barley in the target interval at the sub-cM level. Since rye diverged later (4.0 MYA ± 0.5) than barley (8.9 MYA ± 0.9) from wheat (Middleton et al., 2014) synteny-based mapping-by-sequencing in rye could take advantage from the recently published reference sequence of wheat (IWGSC, 2018). Indeed, the orthologous segments on chromosomes 5A and 4B observed in the present study provide a promising option to promote the marker saturation of the *Ddw1* locus. This applies for instance to the UGT gene residing in the 831 -kb target interval on chromosome 5A, as the glycosylation of gibberellins provides a mechanisms to control the endogenous GA level (Ostrowski and Jakubowska, 2014). Interestingly and in contrast to the results of the present study, no difference in the expression of UGT gene TraesCS5A02G543000 has been reported between *Rht12* and *rht12* lines in wheat (Sun et al., 2019).

Massive Analysis of cDNA Ends here served as a straightforward, powerful yet affordable and easily validated NGS technology for the discovering of new *Ddw1* markers in a species, where a high-quality reference genome sequence is not yet available. The novel gene-based markers we describe here enabled to narrow down the genetic interval of the *Ddw1* region and serve as starting points for high-resolution mapping. In contrast to the study on *Rht12* in wheat (Sun et al., 2019), fine mapping of *Ddw1* in the out-breeding rye was not affected by low recombination rates. As a consequence and based on the wheat RefSeq v1.0, *Rht12* was mapped within a 11.21-Mb segment on chromosome 5A (Sun et al., 2019), while in the present study *Ddw1* could be located in a 831-kb interval residing within this large 5A segment. The markers *MACE3* and *MACE4* rest upon genetic diversity between semi-dwarf and tall genotypes in *ScGA2ox12*, which enabled us to genetically investigate the relationship of this gene and *Ddw1*. Noteworthy, a lack of sequence diversity in TraesCS5A02G543100 between *Rht12* and *rht12* lines excludes TraesCS5A02G543100 as a candidate gene of *Rht12* (Sun et al., 2019). Supposing that the increased expression of TraesCS5A02G543100 is causal for the dwarf phenotype in wheat, two alternatives have been discussed how *Rht12* might control TraesCS5A02G543100, either as a gain-of-function mutant that promotes the expression of the GA2ox gene or as a loss-of-function mutant which suppresses TraesCS5A02G543100 in tall genotypes (Sun et al., 2019). As the *Rht12* locus in wheat includes a 10.73-Mb fragment deletion distal of the marker *W5AC211*, a gain-of-function mutant is proposed to map in a 483-kb region proximal of *W5AC211*, while a loss-of-function could be explained, if *rht12* maps in that 5AL segment, which is deleted in semi-dwarf genotypes distal of *W5AC211* (Sun et al., 2019).

TraesCS5A02G543100 is the updated version of the gene model TraesCS5A01G543100, that was named *TaGA2ox-A14* in the study of Sun et al. (2019). In the present study we have re-named this gene as *TaGA2ox-A12*, following a proposal by Pearce et al. (2015) who classified the *T. aestivum* survey sequence Traes_4BL_57623F302 (Ensemble plants v.25) as a *GA2ox12* gene. We propose to apply the suggestions of Pearce et al. (2015) to the 2-ODDs genes of rye and wheat rather than to continue the index by Sun et al. (2019), the latter of which may give rise to confusion with 2-ODDs genes in both species. Traes_4BL_57623F302 is designated TraesCS4B02G376200 in the newest wheat genome assembly, from the IWGSC (2018). Following this system, the orthologs of *ScGA2ox12*, TraesCS5A02G543100 and TraesCS4B02G376200, would be named *TaGA2ox-A12* and *TaGA2ox-B12*, respectively. The perfect micro-colinearity between the *Ddw1* locus and part of the *Rht12* locus on wheat chromosome 5A, the dominant mode of inheritance as well as the upregulation of both, *ScGA2ox12* and *TaGA2ox-A12* in semi-dwarf rye and wheat genotypes support the assumption, that *Ddw1* and *Rht12* are functionally equivalent one-to-one orthologs (Börner et al., 1996). The close linkage between *Ddw1* and *ScGA2ox12*, thus, suggests a localization of *Ddw1* and *Rht12* in the 531-kb region delimited by the marker loci *c26102* and *W5AC211*, respectively. Within this segment two gene models, TraesCS5A02G543000 and TraesCS5A02G543100, have been predicted in wheat. However, it needs to be considered that the reference wheat genome sequence was established for the tall bread wheat variety “Chinese Spring” (IWGSC, 2018), which carries the recessive *rht12* allele (Sun et al., 2019) and, thus, might not contain the target gene. Drawbacks like this are well known from previous comparative studies where disruption of colinearity as well as non-conserved gene content were observed between related species or even within the same species, which asks in the final step of map-based cloning projects for a genomic library of a genotype that contains the gene of interest (Isidore et al., 2005). With respect to *Ddw1* this methodical consideration is strengthened by the observation, that the gene content was found dissimilar between the 5A and 4B segments in bread wheat, which hampers the prediction of the gene content and order in the orthologous region on rye chromosome 5R. Notably, *ScGA2ox12* revealed a closer evolutionary relationship to *TaGA2ox-B12* as compared to *TaGA2ox-A12*.

The novel markers now enable the tracking of *Ddw1* in rye breeding programs with a precision not feasible before. Breeding of semi-dwarf population varieties is challenged by a low frequency of tall plants, which may persist in populations and increase in frequency if not removed in each generation (McLeod et al., 2000). As demonstrated in the present study, the gene-derived *Ddw1* markers enable to identify *Ddw1* in independent genetic backgrounds. Genetic fingerprinting using flanking co-dominant *Ddw1* markers will support the identification of the recessive *ddw1* allele and, thus, increase the efficiency in breeding of semi-dwarf rye varieties. To conclude, the novel markers enable the introgression of *Ddw1* in elite rye germplasm with unprecedented precision and accelerate the progress in breeding of semi-dwarf rye compared to selection on phenotypes alone.

We have validated the localization of *Ddw1* on the distal end of the long arm of rye chromosome 5R. The previously reported linkage between *Ddw1* and *ct2* as well as *An5* (Smirnov and Sosnichina, 1984) could be confirmed and supplemented by molecular markers. The genetic mapping of *An5* to the distal end of the long arm of chromosome 5R further specifies the localization of a gene of the anthocyanin biosynthesis pathway in rye, which has been assigned to chromosome 5R using trisomics of the anthocyaninless rye cv. “Esto” (Melz and Thiele, 1990). The recombination frequency between *Ddw1* and *Hp* estimated in the present study compares well with previous results (Korzun et al., 1996). However, contradictory orders in both maps ask for further research to precisely determine the position of *Hp* proximal or distal from *Ddw1*.

The markers developed in the present study facilitated comparative mapping of *Ddw1* in rye and Triticale. In Triticale breeding, *Ddw1* served to improve lodging resistance (Wolski and Gryka, 1996). The estimated size of the *rPt-507664*/*rPt-508266* interval flanking the *Ddw1* locus are congruent in rye and Triticale and provides empirical evidence, that a major QTL for PH and biomass yield in Triticale (Alheit et al., 2014; Liu et al., 2014; Würschum et al., 2014a,b) most likely is governed by *Ddw1*. In contrast, a QTL on chromosome 5R with minor effects on PH in rye (Miedaner et al., 2012) can be excluded as being allelic to *Ddw1*, as the map position of this QTL and *Ddw1* do not correspond. This conclusion is supported by the EST-derived SSR marker *Xscm312*, which has been mapped on the distal end of the long arm of chromosome 5R (Hackauf et al., 2009; Martis et al., 2013). In the present study, we estimated the position of this marker 19.7 cM distal of *Ddw1* marker *MACE3*. However, in the Lo115-NxLo117-N population *Xscm312* mapped 293 cM apart from PH QTL#8, which has been assumed to be allelic to *Ddw1* (Miedaner et al., 2012). To conclude, the novel markers will improve and promote genomics-assisted breeding and research on *Ddw1* in rye as well as in Triticale.

Massive Analysis of cDNA Ends enabled both, mapping and quantifying transcriptomes to better understand the genetic control of gene expression in semi-dwarf rye as well as the relationship between genotype and phenotype. Expression pattern, chromosomal localization and linkage analysis identified *ScGA2ox12* associated with *Ddw1*. Next to *TaGA2ox-A12*, *ScGA2ox12* is closely related to *OsGA2ox6* and *TaGA2ox-A9*, which have recently been reported to be associated with semi-dwarfism in rice (Lo et al., 2017) and wheat (Ford et al., 2018). *Rht18* is a dominant and GA responsive mutant and genetically as well as functionally distinct from the widely used GA-insensitive semi-dwarfing genes *Rht-B1b* and *Rht-D1b* in wheat (Ford et al., 2018). The increased expression of *ScGA2ox12* observed in semi-dwarf rye corresponds with the observed expression pattern of *TaGA2ox-A9* (Ford et al., 2018) and *TaGA2ox-A12* (Sun et al., 2019) in wheat and implicates, that *Ddw1* is a dominant gain-of-function mutant, which acts similar to *Rht18* and *Rht12* and which may cause transcriptional activation of *ScGA2ox12*. In transgenic *A. thaliana* lines, up-regulation of *AtGA2ox8* is affected by overexpression of the transcription factor *GhMADS14* (Zhou et al., 2014). The Lo7 rye genome survey contig 1361334 carries the putative ortholog of *GhMADS14*, Sc1Loc00285716.3 (71% identity, *E*-value 1.00 × E-53), which maps to chromosome 1R and can, thus, be excluded as a candidate gene for *Ddw1*.

As confirmed in the present study, *Ddw1* maps to the distal part of the long arm of chromosome 5R, which probably might not compromise a map-based cloning approach for a further molecular characterization of *Ddw1*. We observed a structural alteration in transcript sequences associated with the *ScGA2ox12* gene. Such structural variants may result from splicing of the same gene or post-transcriptional processes (Manning and Cooper, 2017). Alternatively, the transcript co-segregating with *Ddw1* may be encoded by a paralog of the GA2-oxidase gene, which gained a novel function subsequent of a gene duplication, while the original copy retained the ancestral function. Examples where neofunctionalization after duplication has likely contributed to duplicate retention have been reported in plants (Panchy et al., 2016). The recombination observed between both transcripts suggests, that at least two *ScGA2ox12* genes are residing at the *Ddw1* locus. In the *Ddw1* orthologous segment on wheat chromosome 4B, two adjacent GA2 oxidase gene models, TraesCS4B02G376100 and TraesCS4B02G376200, have been predicted as well (Supplementary Table 6). This observation supports the assumption, that gene duplication and neofunctionalization provides the basis for the structural variation between both rye transcripts. Further research including high-resolution mapping and physical map construction of the *Ddw1* locus is in progress to validate this hypothesis. Furthermore, field phenotyping of semi-dwarf rye hybrids is necessary to illustrate, whether the phenotypic effects of *Ddw1* contribute to a better fitness of semi-dwarf rye, e.g., under abiotic stress conditions or via an optimized allocation of dry matter to the grain. The acquisition of such novel adaptive functions may explain the maintenance of both duplicates in the evolution of semi-dwarf rye carrying *Ddw1*.

## Conclusion

Plant architectural traits such as PH are important targets to improve the yield potential of crops. The characterization of *Ddw1* presented in this study expands the genetic options to improve PH and lodging tolerance in rye. The novel markers are valuable means for breeding semi-dwarf rye varieties, as they allow to efficiently track the GA-sensitive dwarfing gene *Ddw1* in breeding programs and to establish genotypes homozygous for this dwarfing gene. Under a changing climate, the systematic exploitation of heterosis together with a genetically optimized allocation of dry matter to the grain offers an approach to increase rye productivity, which is substantially different from current methods in the genetic improvement of rye and wheat. Recent progress concerning genomic resources available for rye open novel opportunities for in-depth analysis of *Ddw1*, which will increase our understanding of GA function in cereals as a fundamental step in exploiting plant genetic diversity for breeding and improving crop species.

## Data Availability

All datasets for this study are included in the manuscript and the Supplementary File.

## Author Contributions

The work presented here was carried out in collaboration between all authors. BH and GM defined the research theme. BH conceived the design of this study, coordinated the experiments, and supervised the project. AV, BH, and GM developed the plant materials. E-MB, BR, and NK accomplished the transcript profiling. E-MB analyzed the rye GA biosynthesis and signaling pathway, and performed the phylogenetic analysis. E-MB, JP, KS, and NT conducted the genotyping. BH and DS established the genetic linkage maps. PW discussed the analyses and interpretation of the results. BH and E-MB interpreted the results and wrote the manuscript. All authors have read and approved the final version of the manuscript.

## Conflict of Interest Statement

BR, NK, and PW are employed by the company GenXPro GmbH. DS is employed at HYBRO Saatzucht GmbH & Co. KG. GM is employed by Nordic Seed Germany GmbH. JP is employed by the company TraitGenetics GmbH. The companies have commercial interest in the results for application in variety development and for the provision of molecular genetic services. The remaining authors declare that the research was conducted in the absence of any commercial or financial relationships that could be construed as a potential conflict of interest.

## Footnotes

1 ftp://ftpmips.helmholtz-muenchen.de/plants/brachypodium/v1.2

2 http://www.traitgenetics.com
