# Supplementary material for: Gene Expression Profiling and Fine Mapping Identifies a Gibberellin 2-Oxidase Gene Co-segregating With the Dominant Dwarfing Gene Ddw1 in Rye (Secale cereale L.)
Source: Front Plant Sci. 2019 Jul 3;10:857. doi: 10.3389/fpls.2019.00857 (PMC6616298; doi:10.3389/fpls.2019.00857)
Supplement: Supplementary file 8 [file Data_Sheet_1.pdf]

Supplementary Material

Gene Expression Profiling and Fine Mapping Identifies a Gibberellin 2-Oxidase Gene Co-segregating With the Dominant Dwarfing Gene *Ddw1* in Rye (*Secale cereale* L.)

Eva-Maria Braun, Natalia Tsvetkova, Dörthe Siekmann, Konrad Schwefel, Nicolas Krezdorn, Jörg Plieske, Björn Rotter, Peter Winter, Gilbert Melz, Martin Ganal, Andrzej Kilian, Anatoly V. Voylokov, and Bernd Hackauf*

*** Correspondence:** Corresponding Author: bernd.hackauf@julius-kuehn.de

1 Supplementary Data

Supplementary Material should be uploaded separately on submission. Please include any supplementary data, figures and/or tables. All supplementary files are deposited to FigShare for permanent storage and receive a DOI.

Supplementary material is not typeset so please ensure that all information is clearly presented, the appropriate caption is included in the file and not in the manuscript, and that the style conforms to the rest of the article.

2 Supplementary Figures and Tables

2.1 Supplementary Figures

Phase	Process	Description
1	**R1620** *ddw1ddw1* x **R347/1** *Ddw1Ddw1*	Crossing of a tall wild type with a semi-dwarf mutant plant
2	**F1** ⓧ *Ddw1ddw1*	Selfing
3	**F2 SP** ⓧ *Ddw1Ddw1* *Ddw1/ddw1* *ddw1ddw1*	Selfing of semi-dwarf F2 SP
4	**F3 L** ⓧ *Ddw1Ddw1* *Ddw1/ddw1* *ddw1ddw1*	Selfing of semi-dwarf F3 SP
5	**F4 L** ⓧ *Ddw1Ddw1* *Ddw1/ddw1* *ddw1ddw1*	Marker-assisted selection and selfing of *Ddw1ddw1* F4 SP
6	**F5 L** ⓧ *Ddw1Ddw1* *Ddw1/ddw1* *ddw1ddw1*	Population of recombinant inbred lines,<br>Marker assisted selection and selfing of F5 SP
7	**F6 L**ⓧ *Ddw1Ddw1* **F6 L**ⓧ *ddw1ddw1*	Selfing of F6 SP
8	**F7 NIB** *Ddw1Ddw1* **F7 NIB** *ddw1ddw1*	Tissue sampling and expression profiling

Abbreviations and symbols

FxSP, FxL	Single plant respectively line in the xth generation of selfing
Ddw1Ddw1, Ddw1ddw1, ddw1ddw1	Dominant dwarf1 (*Ddw1*) gene with homozygous mutant, heterozygous mutant, and homozygous wild type allele
NIB	Near isogenic bulk
/	Progeny segregating for the *Ddw1* gene
ⓧ	Selfing

Supplementary Figure 1. Development of a recombinant inbred line (RIL) population segregating for *Ddw1* and pairs of near isogenic inbred bulks (NIB) that are carriers or non-carriers of the *Ddw1* gene by phenotypic and marker-assisted selection. Selected progenies are highlighted in red.

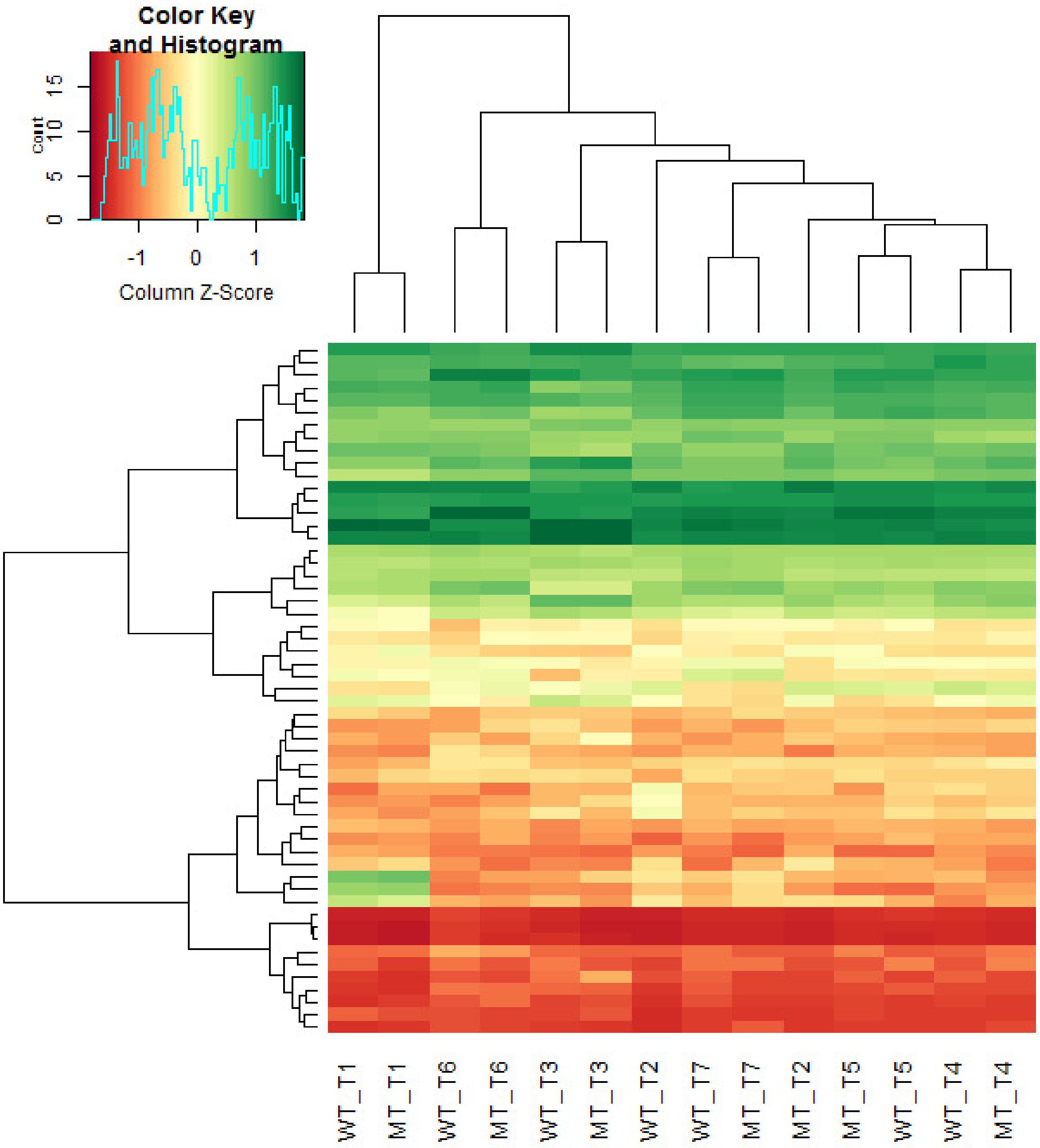

Supplementary Figure 2: Hierarchical clustering heatmap of rye homologs of rice genes controlling germination in different tissues of semi-dwarf and tall rye genotypes. Each row represents a contig and each column represents a defined tissue and genotype. The z-score of fold-change values for each sample were subjected to hierarchical clustering using standard correlation. The color scale ranges from negative (red) to positive z-scores (green) explaining the deviance of mean expression. WT: normal NIL, MT: semi-dwarf NIB, T1: **Root and caryopses** EC07, T2: **Coleoptile** EC07, T3: **Leaf** EC12, T4: **Stem** EC29, T5: **Stem** EC37, T6: **Stem** EC51, T7: **Ear** EC51.

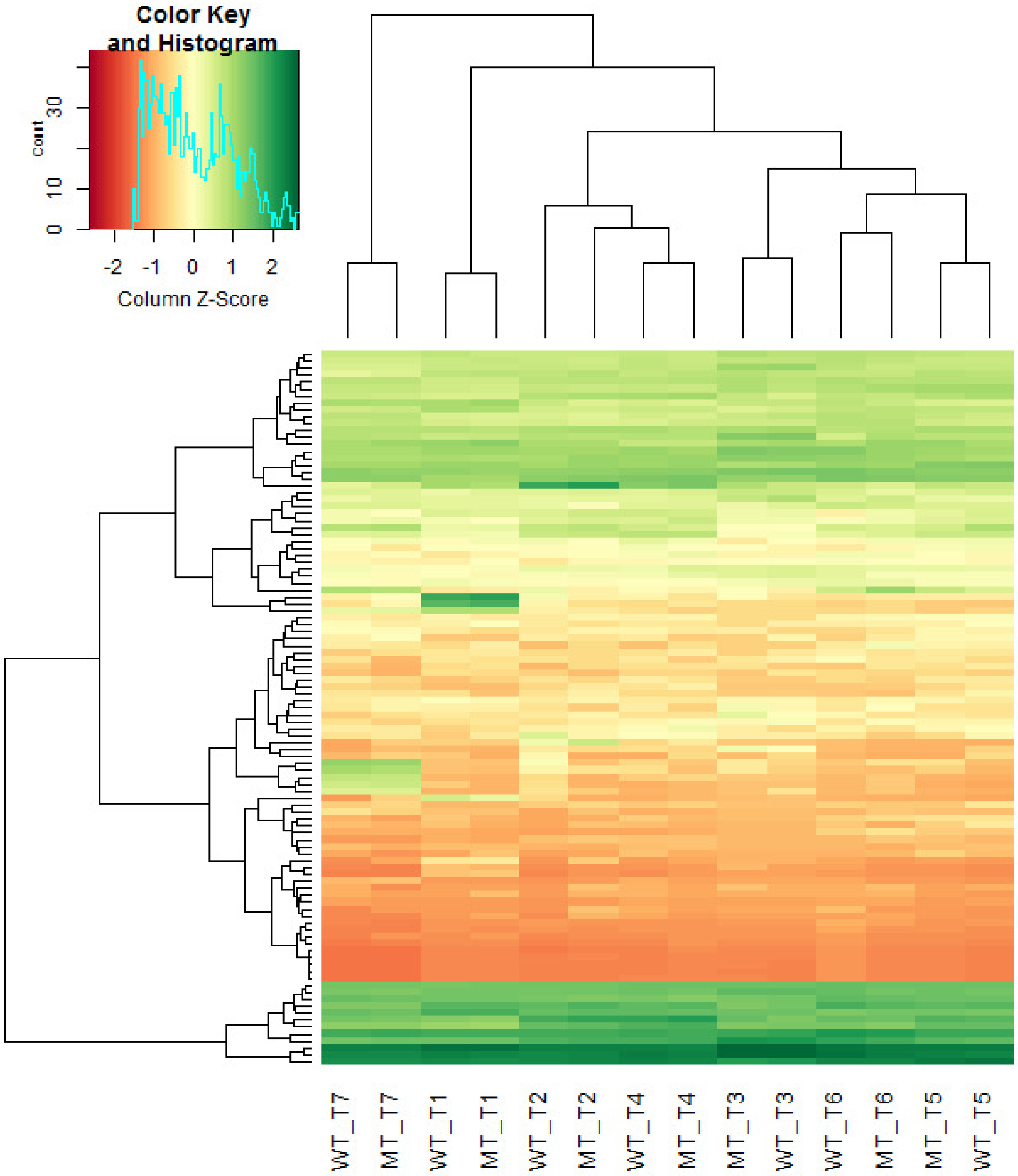

Supplementary Figure 3: Hierarchical clustering heatmap of rye homologs of rice genes controlling the development of seedlings and shoots in different tissues of semi-dwarf and tall rye genotypes. Each row represents a contig and each column represents a defined tissue and genotype. The z-score of fold-change values for each sample were subjected to hierarchical clustering using standard correlation. The color scale ranges from negative (red) to positive z-scores (green) explaining the deviance of mean expression. WT: normal NIL, MT: semi-dwarf NIB, T1: **Root and caryopses** EC07, T2: **Coleoptile** EC07, T3: **Leaf** EC12, T4: **Stem** EC29, T5: **Stem** EC37, T6: **Stem** EC51, T7: **Ear** EC51.

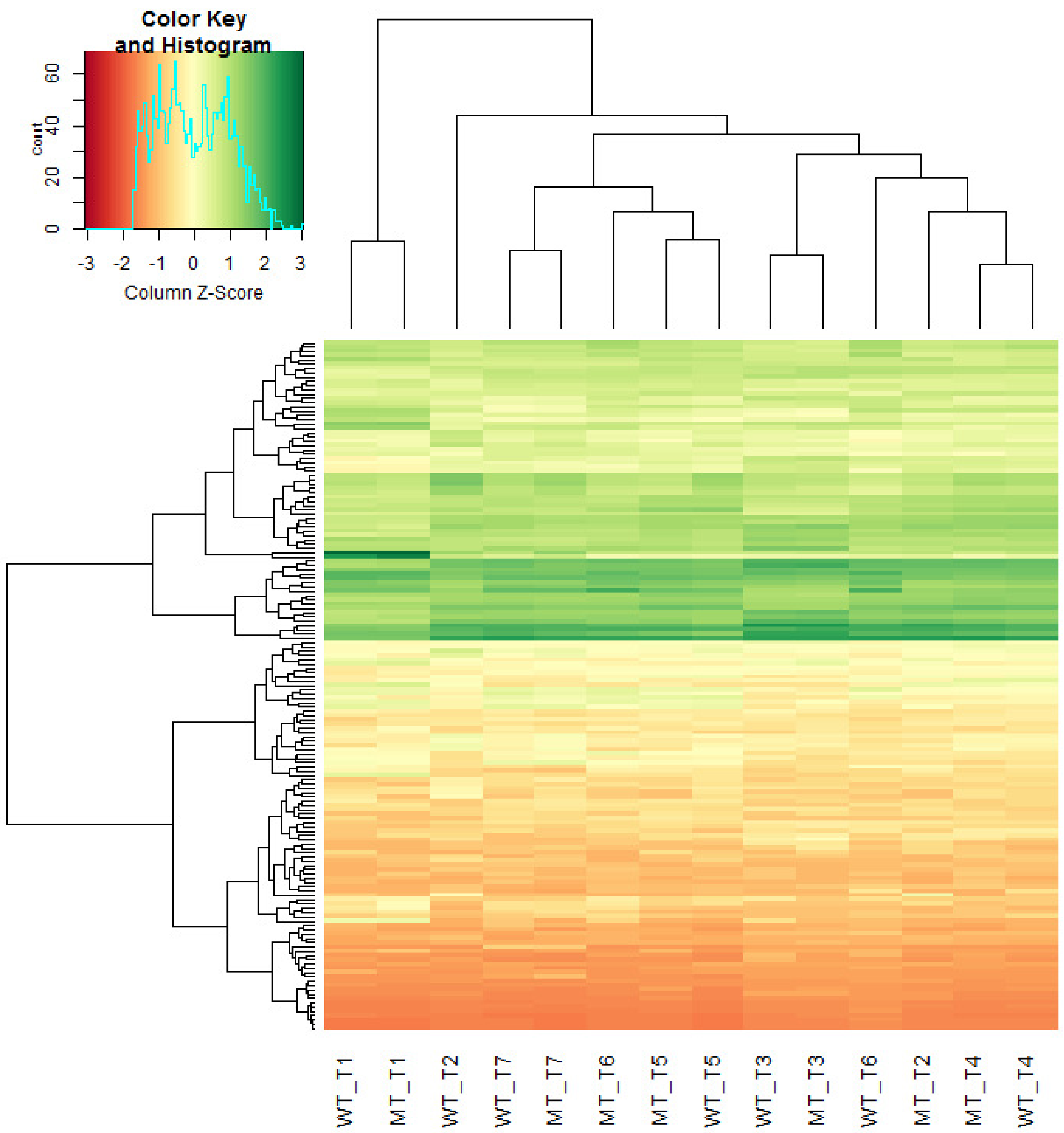

Supplementary Figure 4: Hierarchical clustering heatmap of rye homologs of rice genes controlling source/sink activity in different tissues of semi-dwarf and tall rye genotypes. Each row represents a contig and each column represents a defined tissue and genotype. The z-score of fold-change values for each sample were subjected to hierarchical clustering using standard correlation. The color scale ranges from negative (red) to positive z-scores (green) explaining the deviance of mean expression. WT: normal NIL, MT: semi-dwarf NIB, T1: **Root and caryopses** EC07, T2: **Coleoptile** EC07, T3: **Leaf** EC12, T4: **Stem** EC29, T5: **Stem** EC37, T6: **Stem** EC51, T7: **Ear** EC51.

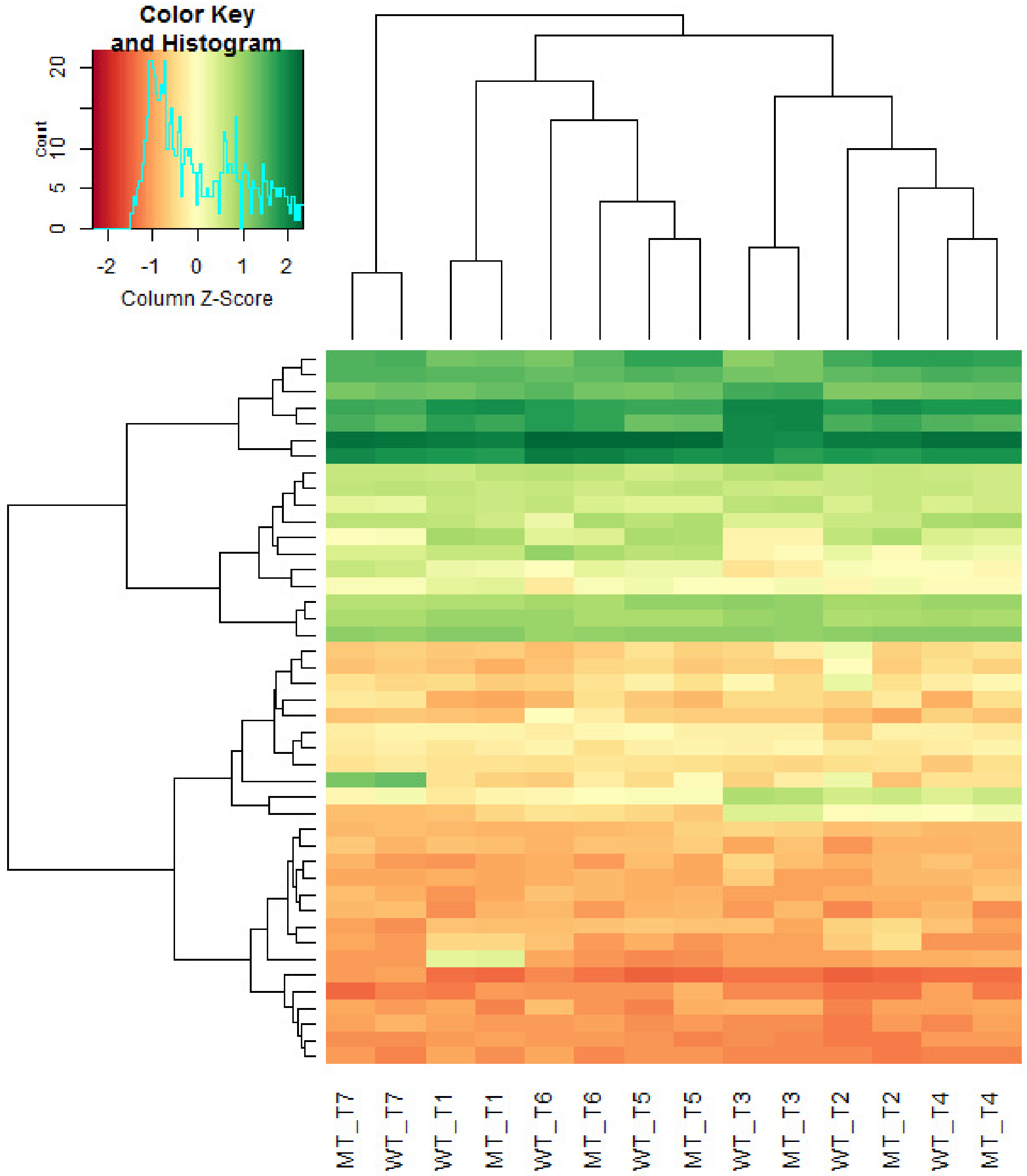

Supplementary Figure 5: Hierarchical clustering heatmap of rye homologs of rice genes controlling flowering in different tissues of semi-dwarf and tall rye genotypes. Each row represents a contig and each column represents a defined tissue and genotype. The z-score of fold-change values for each sample were subjected to hierarchical clustering using standard correlation. The color scale ranges from negative (red) to positive z-scores (green) explaining the deviance of mean expression. WT: normal NIL, MT: semi-dwarf NIB, T1: **Root and caryopses** EC07, T2: **Coleoptile** EC07, T3: **Leaf** EC12, T4: **Stem** EC29, T5: **Stem** EC37, T6: **Stem** EC51, T7: **Ear** EC51.

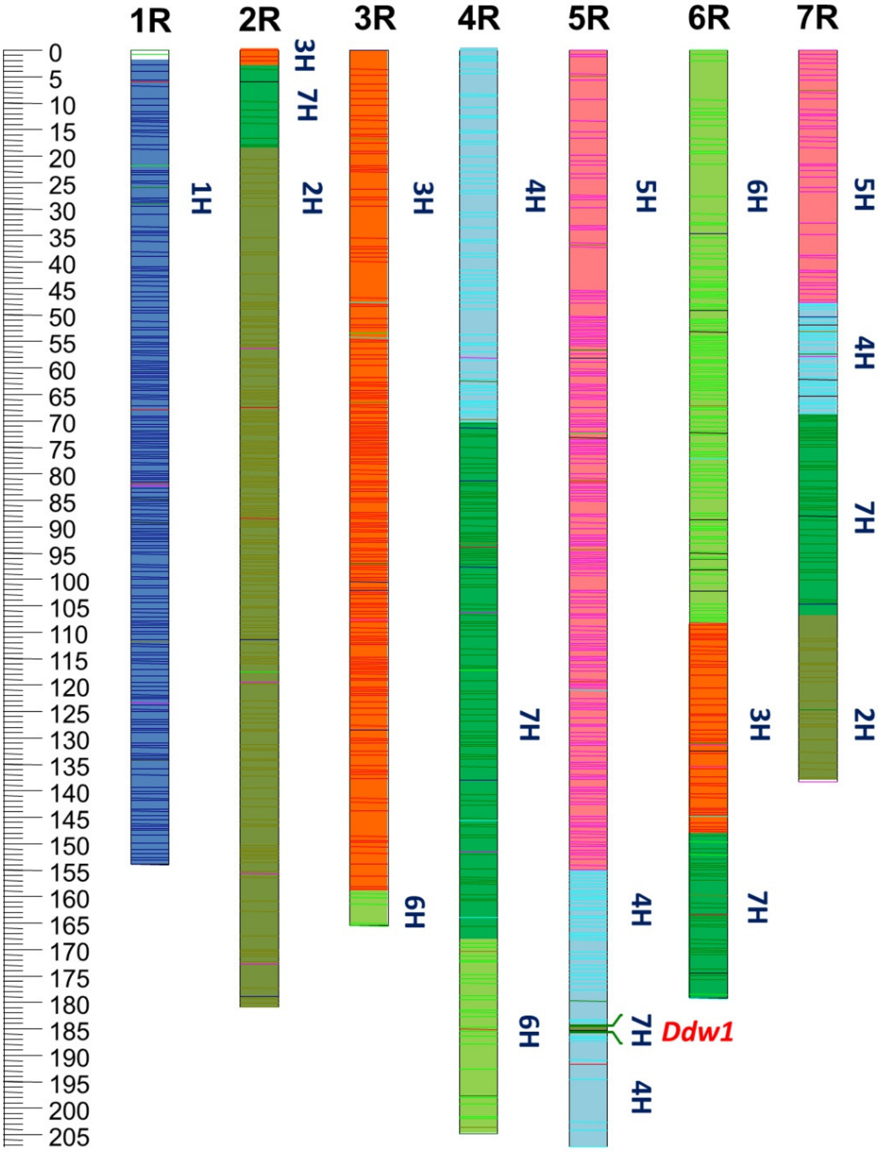

Supplementary Figure 6. Integration of MACE assemblies in the high-density map of rye and relationship of the seven rye chromosomes to the homeologous chromosomes of barley.

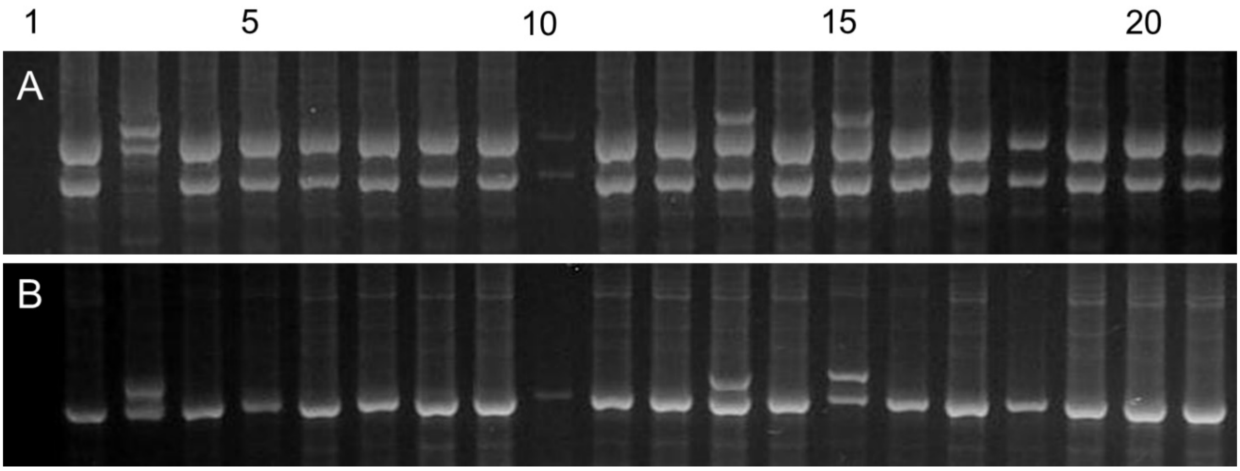

Supplementary Figure 7. Chromosomal localization of the Ddw1 markers (A) tcos4366 and (B) tcos1137 using monosomic disomic wheat/rye translocation addition and translocation lines. Lanes: (1) no template control, (2) *T. aestivum* cv. 'Chinese Spring', (3) *S. cereale* cv. 'Imperial', (4) 21"+1R", (5) 21"+1RL", (6) 21"+2R´/21"+2R´+2RL´, (7) 21"+2RL", (8) 21"+3R", (9) 21"+3RS", (10) 21"+4R", (11) 21"+4RS", (12) 21"+4RL", (13) 21"+5R", (14) 21"+5RS", (15) 21"+5RL", (16) 21"+6R", (17) 21"+6RS", (18) 21"+6RL", (19) 21"+7R", (20) 21"+7RS", (21) 21"+7RL".

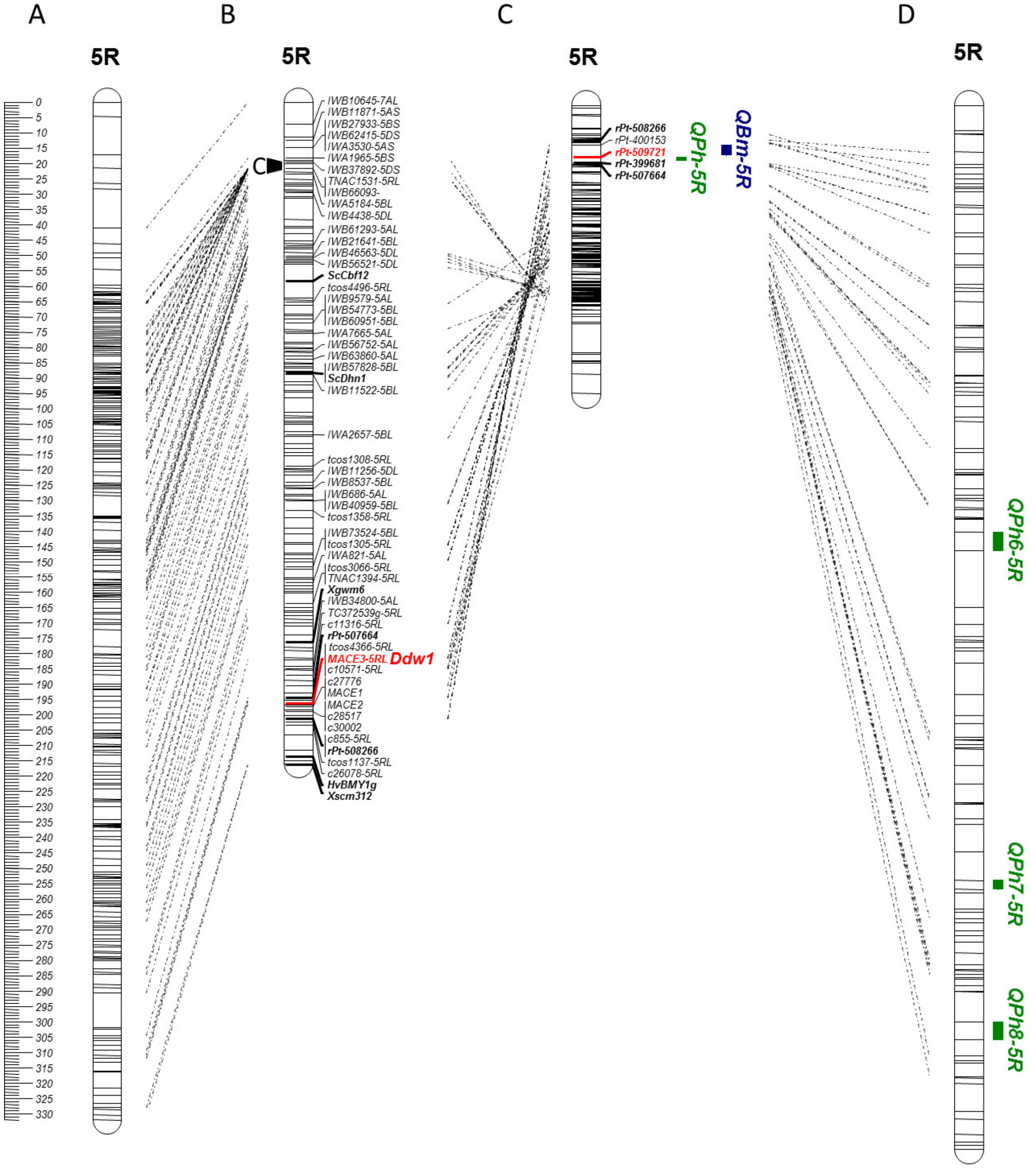

Supplementary Figure 8. Comparative mapping of *Ddw1* in rye and Triticale. (A) Integrated linkage map of chromosome 5R according to Martis et al. (2013). (B) Linkage map of chromosome 5R established in a RIL population from the cross 'L2039-N' x DH'. The estimated position of the centromere is indicated by the symbol C. (C) Linkage map of chromosome 5R generated in the Triticale population EAW78 (according to Ahlheit et al. 2011, 2014). (D) Linkage map of chromosome 5R constructed for the rye population Lo115-NxLo117-N (according to Miedaner et al. 2012). Common markers between individual maps are connected by dotted lines. The positions of the

markers are given in cM. The vertical bars and QTL symbols indicate the position of the following quantitative traits: QBm: biomass yield, QPh: plant height.

2.2 Supplementary Tables

Supplementary Table 1: PCR primers and conditions for the 5RL sequence-tagged site markers.
Supplementary Table 2: Segregation data from *Ddw1* genotypes in $F_{4:5}$ families from the cross R1620 x R347/1.
Supplementary Table 3: Rye gibberellin biosynthesis and signaling genes.
Supplementary Table 4: Gene expression profiles of mutant and wildtype *Ddw1* genotypes in rye.
Supplementary Table 5: Rye orthologs of cloned rice QTL.
Supplementary Table 6: The orthologous *Ddw1* segment on wheat chromosome 4B.
Supplementary Table 7: High-density linkage map of chromosome 5R in the RIL population L2039-NxDH.

frontiers
